# Stattic enhances the anti-tumor activity of AZD4547 in LUSC by blocking STAT3/RRM2-mediated DNA repair and inducing ROS-driven mitochondrial dysfunction

**DOI:** 10.1038/s41419-026-08848-1

**Published:** 2026-05-19

**Authors:** Jiehong Wang, Yue Hao, Ke Wang, Naiyan Lu, Xue Zhu, Jing Fang, Hong Shu, Xun Wang

**Affiliations:** 1 Department of Pulmonary and Critical Care Medicine, Affiliated Hospital of Jiangnan University, Wuxi School of Medicine, Jiangnan University, Wuxi, Jiangsu Province China; 2 National Health Commission Key Laboratory of Nuclear Medicine, Jiangsu Key Laboratory of Molecular Nuclear Medicine, Jiangsu Institute of Nuclear Medicine, Wuxi, Jiangsu Province China; 3 Department of Respiratory Medicine, Affiliated Jinling Hospital, Medical School of Nanjing University, Nanjing, Jiangsu Province China; 4 Department of Radiopharmaceuticals, School of Pharmacy, Nanjing Medical University, Nanjing, Jiangsu Province China; 5 School of Food Science and Technology, Jiangnan University, Wuxi, Jiangsu Province China; 6 Department of Clinical Laboratory, Guangxi Medical University Cancer Hospital, Nanning, Guangxi Province China

**Keywords:** Non-small-cell lung cancer, Molecular biology

## Abstract

Fibroblast growth factor receptor (FGFR) gene alterations are relatively frequent in lung squamous cell carcinoma (LUSC) and represent potential targets for therapy with FGFR inhibitors. However, FGFR inhibitor monotherapy is often undermined by compensatory survival pathways. In this study, a combinatorial therapeutic approach using the STAT3 inhibitor Stattic together with the pan-FGFR inhibitor AZD4547 for FGFR1-positive LUSC were assessed. The results showed that AZD4547 suppresses FGFR1 phosphorylation, triggers IL-6/STAT3 activation and induces RRM2-dependent DNA repair, limiting single-agent efficacy. Combining AZD4547 with the STAT3 inhibitor Stattic synergistically impaired cell proliferation, colony formation, and survival, and markedly enhanced apoptosis in vitro and in vivo. Mechanistically, dual inhibition disrupted the STAT3/RRM2 axis, promoting DNA damage and simultaneously provoking ROS-induced mitochondrial dysfunction. These findings nominate concurrent FGFR1 and STAT3 blockade as a promising therapeutic approach for FGFR1-positive lung squamous cell carcinomas.

## Introduction

Non-small cell lung cancer (NSCLC), primarily categorized into two major histological subtypes of adenocarcinoma and squamous cell carcinoma, remains the leading cause of cancer-related mortality worldwide [1]. Compared with those with the squamous cell carcinoma, patients with adenocarcinoma have more treatment options, including several tyrosine kinase inhibitors, immunotherapy, and various combination strategies guided by precise next-generation sequencing [2]. Squamous cell carcinoma, which accounts for 20–30% of non-small cell lung cancer cases, is a histological subtype linked to inferior outcomes, with treatment options remaining limited to date [3].

Compared with lung adenocarcinoma, lung squamous cell carcinoma harbors a substantially lower frequency of clinically actionable driver mutations, which has limited the development and success of targeted therapies in this subtype. While lung adenocarcinoma patients often benefit from well-established targeted treatments against alterations such as EGFR, ALK, or ROS1, actionable genomic events in lung squamous cell carcinoma remain relatively infrequent and heterogeneous [4]. Moreover, targeted therapies in lung squamous cell carcinoma have shown less consistent clinical efficacy, with variable response durability and a high propensity for intrinsic or acquired resistance [5]. These biological and therapeutic challenges underscore the unmet clinical need for improved treatment strategies in lung squamous cell carcinoma and highlight the importance of identifying novel molecular vulnerabilities beyond canonical driver mutations. Currently, only a few immune checkpoint inhibitors combined with chemotherapy, such as Nivolumab or Ipilimumab, are currently recommended in lung squamous cell carcinoma clinical practice.

Fibroblast growth factor receptors (FGFRs) are a family of receptor tyrosine kinases that are involved in the regulation of cell proliferation, survival, and development. The FGFR1-4 genes of the fibroblast growth factor (FGF) family encode four distinct tyrosine kinase receptors [6]. FGFR alterations represent key targetable genetic drivers underlying the pathogenesis of lung squamous cell carcinoma (LUSC). Emerging evidence indicates that FGFR2 and FGFR3 mutations occur in roughly 6% of lung squamous cell carcinomas and exhibit oncogenic properties in cellular transformation assays and experimental models [7]. A study involving 143 treatment-naive patients with early- to mid-stage lung squamous cell carcinoma reported that approximately 17% harbored FGFR mutations (including both somatic and germline variants). Notably, patients with somatic mutations exhibited significantly worse prognosis, suggesting that FGFR alterations represent an independent adverse prognostic factor [8]. FGFR signaling can influence cancer-related pathways such as PI3K/AKT and signal transducer and activator of transcription (STAT), thereby influencing cell proliferation, metabolism, migration, and cell cycle progression [9]. Therefore, targeting FGFRs holds promise as a potential therapeutic approach for LUSC patients [10]. Recently, a series of FGFR-target drugs have been developed [11]. As a small-molecule FGFR kinase inhibitor, AZD4547 effectively inhibits recombinant FGFR kinase activity in vitro, suppresses FGFR signaling and cell proliferation in tumor cell lines with dysregulated FGFR expression, and demonstrates notable antitumor activity in FGFR-positive human tumor xenograft models [12]. However, in a clinical study evaluating AZD4547 in previously treated patients with advanced LUSC harboring FGFR1 amplification, only one patient achieved a partial response, indicating limited antitumor activity [13]. The heterogeneous nature of these tumors, characterized by co-occurring mutations and marked variability in gene expression within the 8p11 amplicon, likely contributes to the limited efficacy of FGFR inhibitors in this setting [13]. Similarly, in the Lung-MAP sub-study S1400D, only two patients achieved partial responses, demonstrating limited clinical activity [14]. This study concluded that AZD4547 has limited efficacy in this cohort predominantly characterized by FGFR1/3 amplification [14]. Therefore, the modest antitumor activity observed with AZD4547 monotherapy necessitates therapeutic optimization [15], and combined therapy such as dual inhibition of parallel signaling cascades may be an effective strategy [16–18].

STAT3 (Signal Transducer and Activator of Transcription 3) is a pivotal transcription factor that drives cancer development and progression [19]. By orchestrating processes such as cell proliferation, apoptosis resistance, metabolic reprogramming, and immune evasion, STAT3 facilitates tumor growth and metastasis [20, 21]. Its persistent activation correlates with poor prognosis across various cancer types, making STAT3 a compelling target for therapeutic intervention. Mechanistic studies suggest that STAT3 activation can bypass inhibited FGFR signaling, promoting tumor survival through alternative pathways [22]. Current FGFR-targeted therapies often show limited efficacy, partly due to compensatory activation of parallel signaling cascades, such as the JAK/STAT and MAPK pathways [23]. Recently, some researchers also highlighted the interplay between FGFR activation and the JAK/STAT pathway, emphasizing that compensatory signaling through parallel pathways may limit the effectiveness of FGFR-targeted therapies [24]. These observations highlight the need to understand the interplay between STAT3 and FGFR inhibition, as co-targeting STAT3 may improve the therapeutic efficacy of FGFR inhibitors in LUSC. Preclinical studies have demonstrated that the V561M gatekeeper mutation in FGFR1 can compromise the efficacy of AZD4547 by activating STAT3 in lung squamous cell carcinoma, and that STAT3 knockdown may restore sensitivity to FGFR1 inhibitors [25]. Despite these findings, comprehensive mechanistic insights and corroborating evidence remain limited.

Emerging data indicate that STAT3 plays a pivotal role in modulating the response to AZD4547; however, whether STAT3 loss can enhance AZD4547 efficacy in LUSC has yet to be determined. In this study, we investigated the therapeutic potential of combining AZD4547 with the STAT3 inhibitor Stattic, with the aim of identifying novel treatment strategies for patients with LUSC harboring rare FGFR alterations.

## Materials and methods

### Chemicals and reagents

AZD4547 and Stattic were purchased from MedChemExpress (Shanghai, China). Primary antibodies used in this study included: p-FGFR1 (ab173305), FGFR1 (ab76464), FGFR2 (ab109372), FGFR3 (ab133644), FGFR4 (ab178396), p-AKT (ab192623), AKT (ab18785), γ-H2AX (ab81299), p-STAT3 (Y705) (ab267373), and GAPDH (ab8245), all obtained from Abcam (Cambridge, MA, USA). Additional antibodies, including p-FGFR2 (abs140266), p-FGFR3 (abs140268), and p-FGFR4 (abs139979), were obtained from Absin (Shanghai, China). ERK1/2 (AF1051) and p-ERK1 (Thr202/Thr204)/ERK2 (Thr185/Thr187) (AF1891) were purchased from Beyotime (Nantong, China). Antibodies against IL-6 (21865-1-AP), STAT3 (60199-1-Ig, 80149-1-RR), Cytochrome c (10993-1-AP), VDAC (81538-1-R) and RRM2 (11661-1-AP) were sourced from Proteintech (Wuhan, China). Other general reagents were obtained from Sangon (Shanghai, China) and Beyotime (Nantong, China).

### Cell lines and culture

The human lung squamous carcinoma cell line H1703 was obtained from ATCC (American Type Culture Collection, VA, USA). The human lung squamous carcinoma cell line H520 and the human bronchial epithelial cell line BEAS-2B were obtained from the National Collection of Authenticated Cell Cultures (Shanghai, China). Cells were cultured in RPMI-1640 medium supplemented with 10% (v/v) fetal bovine serum (FBS) and 1% penicillin/streptomycin (P/S) in a humidified atmosphere containing 5% CO₂ at 37 °C.

### Cell viability assays

Cells were seeded into 96-well plates at a density of 1 × 10⁴ cells/well and treated with AZD4547, Stattic, or their combination. After treatment, 5 mg/mL MTT (ST316, Beyotime) was added to each well and incubated for 4 h. Absorbance was measured at 450 nm using a microplate reader (SpectraMax M5, Molecular Devices, CA, USA). Cells were seeded into 6-well plates at 1000 cells/well and cultured, with medium replaced every 3 days. At the endpoint, cells were fixed with methanol for 15 min, stained with 1% crystal violet, and colonies were counted.

### Cell apoptosis assay

Cells were seeded into 24-well plates at 5 × 10⁴ cells/well and treated as indicated. Cells were fixed with 4% paraformaldehyde for 30 min, permeabilized for 7 min at room temperature with enhanced immunostaining permeabilization buffer (P0097, Beyotime), and incubated with TUNEL reaction solution (C1090, Beyotime) for 60 min at 37 °C. Nuclei were counterstained with DAPI, and fluorescence images were acquired using an Olympus IX53 fluorescence microscope.

### Western blot analysis

Cells were lysed in RIPA buffer, and protein concentrations were determined using the BCA Protein Assay Kit (Beyotime). Equal amounts of protein were separated by 15% SDS-PAGE and transferred to PVDF membranes. Membranes were blocked, incubated with primary antibodies at 4 °C overnight, followed by HRP-conjugated secondary antibodies at 37 °C for 2 h. Signals were visualized using an ECL detection kit (Beyotime).

### Molecular docking analysis

Molecular docking was performed to investigate the interactions between receptor proteins and small-molecule ligands. Receptor and ligand structures were prepared using AutoDockTools 1.5.6, including the addition of polar hydrogens, Gasteiger charge assignment, and definition of rotatable bonds. Docking simulations were conducted with AutoDock Vina using default parameters, and the binding poses with the lowest predicted binding energies were selected for further analysis. For docking of AZD4547 with FGFR family proteins, the ZDOCK server (http://zdock.umassmed.edu) was employed to generate potential binding conformations. All docking results were analyzed and visualized using PyMOL (Schrödinger, LLC). Key interactions were annotated: red dashed lines for hydrogen bonds, yellow dashed lines for π-cation interactions, and purple dots indicating aromatic ring centroids.

### Immunofluorescence analysis

Cells were seeded at a density of 1 × 10^4^ cells per well in 24-well plates or confocal dishes and allowed to adhere overnight. Following treatment with the indicated agents, cells were fixed with 4% paraformaldehyde for 30 min at room temperature and permeabilized with 0.1% Triton X-100 for 5 min. Non-specific binding was blocked with 5% goat serum for 1 h at room temperature. Cells were incubated overnight at 4 °C with primary antibodies (specify target and catalog number) diluted in blocking buffer. After three washes with PBS, cells were incubated with appropriate Cy3- or Alexa Fluor 488-conjugated secondary antibodies for 2 h at room temperature in the dark. Nuclei were counterstained with DAPI (10 µg/mL) for 10 min. Fluorescence images were acquired using an Olympus IX53 inverted fluorescence microscope (Olympus, Japan) under consistent exposure settings. Fluorescence intensity per cell was quantified using ImageJ software (NIH, USA).

### Quantitative real-time PCR

Total RNA was extracted using Trizol reagent (Cat. No. R0011, Beyotime, Shanghai, China) according to the manufacturer’s protocol. RNA (1 µg) was reverse transcribed into cDNA using the PrimeScript™ RT-PCR kit (Cat. No. R211, Vazyme, Nanjing, China). qRT-PCR was performed on an ABI 7500 Fast Real-Time PCR System (Thermo Fisher, USA) using SYBR Premix Ex Taq™ (Cat. No. Q712, Vazyme). Primers were designed to specifically amplify target genes (sequences listed in Supplementary Table X), with GAPDH serving as the internal control. Relative gene expression was calculated using the 2⁻ΔΔCt method. All reactions were conducted in triplicate, and data are presented as mean ± SD. Statistical analysis was performed using one-way ANOVA with Tukey’s post hoc test, and *p* < 0.05 was considered statistically significant. Details of the primer sequences and their sources are shown in Supplementary Fig. 1.

### Cell transfection

For gene knockdown or overexpression, cells were transfected with specific siRNAs targeting IL-6, FGFR1, FGFR3, STAT3, or RRM2, or with pcDNA3.1-RRM2 overexpression plasmid (GenePharma, Shanghai, China) using Lipofectamine 3000 (Life Technologies, USA) following the manufacturer’s instructions. Briefly, cells were seeded to reach ~70% confluence at the time of transfection. Forty-eight hours post-transfection, knockdown or overexpression efficiency was verified by Western blotting before subsequent experiments. Detailed sequences and sources of the siRNA are shown in Supplementary Fig. 2.

### Intracellular ROS analysis

Intracellular ROS levels were measured using the DCFH-DA Cellular ROS Detection Kit (Cat. No. C1060, Beyotime, Shanghai, China) according to the manufacturer’s instructions, with minor modifications. Cells were seeded in 24-well plates at a density of 5 × 10^4^ cells per well and cultured overnight. After treatment with the indicated agents, cells were incubated with 10 µm DCFH-DA at 37 °C for 20 min in the dark. Cells were then washed three times with PBS to remove excess probe. Fluorescence images were captured using an Olympus IX53 inverted fluorescence microscope (Olympus, Japan) under identical exposure settings. Fluorescence intensity per cell was quantified using ImageJ software (NIH, USA), and results were normalized to untreated control cells. Hydrogen peroxide (100 µM, 30 min) was used as a positive control.

### Lipid peroxidation assay

Lipid peroxidation was evaluated by measuring malondialdehyde (MDA) levels using the Lipid Peroxidation MDA Assay Kit (Cat. No. ab118970, Abcam, Cambridge, UK). Following treatment, cells were lysed, and protein concentrations were determined by BCA assay. Cell lysates were then incubated with thiobarbituric acid (TBA) reagent at 95 °C for 20 min to form the MDA-TBA adduct. After cooling to room temperature, absorbance was measured at 532 nm using a microplate reader (BioTek Synergy HTX, USA). MDA concentrations were calculated based on a standard curve and normalized to total protein content.

### Superoxide dismutase (SOD) activity

Total SOD activity was measured using the Total Superoxide Dismutase Assay Kit (Cat. No. S0101S, Beyotime, Shanghai, China) according to the manufacturer’s instructions. Briefly, cells were collected after treatment and lysed to obtain protein extracts. Protein concentrations were determined using a BCA assay. Equal amounts of protein were incubated with the SOD detection solution at 37 °C for 30 min. Absorbance was recorded at 450 nm using a microplate reader (BioTek Synergy HTX, USA). SOD activity was calculated according to the kit’s standard curve and normalized to total protein content.

### MitoSO™ Red detection

Mitochondrial superoxide production was assessed using the MitoSO™ Red Mitochondrial Superoxide Indicator Kit (Cat. No. S0061S, Beyotime, Shanghai, China) according to the manufacturer’s instructions, with minor modifications. Briefly, cells were seeded in 24-well plates at a density of 5 × 10^4^ cells per well and cultured overnight. After treatment with the indicated agents, cells were incubated with MitoSO™ Red working solution at 37 °C for 60 min in the dark to specifically detect mitochondrial superoxide. Following incubation, cells were washed three times with PBS to remove excess dye. Fluorescence images were captured using an Olympus IX53 inverted fluorescence microscope (Olympus, Japan) under identical exposure settings. For quantitative analysis, fluorescence intensity per cell was measured using ImageJ software (NIH, USA), and data were normalized to untreated control cells. As a positive control, cells were treated with 100 µM menadione for 30 min to induce mitochondrial superoxide production.

### EdU assay

Cell proliferation was evaluated using the BeyoClick™ EdU Cell Proliferation Kit with Alexa Fluor 488 (Cat. No. C0071S, Beyotime, Shanghai, China) according to the manufacturer’s instructions, with minor modifications. Briefly, cells were seeded in 24-well plates at a density of 5 × 10^4^ cells per well and allowed to adhere overnight. Following treatment with the indicated agents, cells were incubated with 10 µM 5-ethynyl-2’-deoxyuridine (EdU) for 2 h at 37 °C to label newly synthesized DNA. Cells were then washed twice with PBS, fixed with 4% paraformaldehyde for 15 min at room temperature, and permeabilized with 0.5% Triton X-100 for 20 min. EdU incorporation was detected by reaction with the Alexa Fluor 488 azide dye according to the kit protocol. Nuclei were counterstained with Hoechst 33342 (10 µg/mL) for 10 min. Fluorescence images were captured using an Olympus IX53 inverted fluorescence microscope (Olympus, Japan) under consistent exposure settings. The proliferation rate was calculated as the percentage of EdU-positive cells relative to the total number of Hoechst-stained nuclei. At least five randomly selected fields per well were analyzed using ImageJ software (NIH, USA).

### ATP content assay

Cellular ATP levels were determined using a luciferase-based ATP assay kit (Cat. No. S0026, Beyotime, Shanghai, China) following the manufacturer’s instructions with slight modifications. Cells were seeded in 96-well plates at 1 × 10^4^ cells/well and cultured overnight. After treatment, cells were washed twice with ice-cold PBS and lysed with ATP lysis buffer. Lysates were centrifuged at 12,000 × *g* for 5 min at 4 °C, and supernatants were collected. For measurement, 20 µL of lysate was mixed with 100 µL of luciferase/luciferin working solution in white 96-well plates, and luminescence was recorded immediately using a microplate luminometer (BioTek Synergy HTX, USA). ATP concentrations were determined from a standard curve (1 µM–10 nM) and normalized to total protein content measured by a BCA assay (Beyotime, China). Oligomycin-treated cells (1 µM, 2 h) served as a positive control for ATP depletion.

### Mitochondrial membrane potential assay

Mitochondrial membrane potential was measured using the Rhodamine 123 Detection Kit (Cat. No. C2008S, Beyotime, Shanghai, China). Cells were seeded in 6-well plates and cultured to 70–80% confluence. After treatment, cells were washed twice with pre-warmed PBS and incubated with Rhodamine 123 working solution (10 µg/mL) at 37 °C for 60 min in the dark. Excess dye was removed by three washes with PBS. Fluorescence was visualized using an inverted fluorescence microscope (Nikon Eclipse Ti, Japan) with excitation/emission at 507/529 nm. For quantitative analysis, fluorescence intensity was measured using a microplate reader (BioTek Synergy HTX, USA) or by flow cytometry (BD FACSCanto II, USA) when needed. As a positive control for mitochondrial depolarization, cells were treated with 10 µM carbonyl cyanide m-chlorophenyl hydrazone (CCCP) for 30 min.

### Chromatin immunoprecipitation (ChIP)-qPCR

Chromatin immunoprecipitation (ChIP) assays were performed using a commercial Chromatin Immunoprecipitation (ChIP) Assay Kit (Cat. No. P2078, Beyotime, Shanghai, China) with minor modifications. Briefly, cells were cultured to ~80% confluence and cross-linked with 1% formaldehyde for 10 min at room temperature. The cross-linking reaction was quenched with 0.125 M glycine for 5 min, followed by two washes with ice-cold PBS. Cells were harvested, lysed to isolate nuclei, and chromatin was sheared by sonication using a Bioruptor sonicator (Diagenode, Belgium) to obtain DNA fragments averaging 200–500 bp. Sheared chromatin was incubated overnight at 4 °C with either 5 µg anti-STAT3 antibody (STAT3, Cat. No. 80149-1-RR, Proteintech, Wuhan, China) or normal rabbit IgG as a negative control, both pre-bound to protein A/G magnetic beads. Immunoprecipitates were washed extensively, eluted, and reverse cross-linked at 65 °C for 4 h in the presence of proteinase K. DNA was purified using a PCR Clean Up Kit/DNA Purification Kit (Cat. No. D0033, Beyotime, Shanghai, China) according to the manufacturer’s instructions. Quantitative PCR (qPCR) was conducted using SYBR Green Master Mix on a QuantStudio™ 6 Flex Real-Time PCR System (Applied Biosystems, USA). Primers targeting the putative STAT3-binding site within the RRM2 promoter were used. Enrichment of STAT3 binding was calculated as a percentage of input chromatin and normalized to IgG controls. The complete primer sequences as well as the corresponding amplicon genomic regions (coordinates/length) are provided in Supplementary Fig. 3.

### Flow cytometry analysis

Cells were collected and processed for flow cytometric analysis according to the manufacturer’s instructions (Absin Bioscience Inc., Shanghai, China). Briefly, cells were harvested, washed twice with cold phosphate-buffered saline (PBS), and resuspended at an appropriate concentration. For surface marker analysis, cells were incubated with fluorochrome-conjugated antibodies in the dark at 4 °C for 30 min. After incubation, cells were washed twice with PBS to remove unbound antibodies. For apoptosis analysis, cells were stained using the Annexin V-FITC/Propidium Iodide (PI) apoptosis detection kit (Absin) following the manufacturer’s protocol. Briefly, cells were resuspended in binding buffer and incubated with Annexin V-FITC and PI at room temperature in the dark for 15 min. For cell cycle analysis, cells were fixed in 70% cold ethanol at 4 °C overnight, washed with PBS, and then incubated with RNase A and propidium iodide staining solution for 30 min at room temperature in the dark. All samples were analyzed using a flow cytometer (BD FACSCanto II), and at least 10,000 events were collected for each sample. Data were analyzed using FlowJo software. Appropriate isotype controls and unstained controls were included to ensure accurate gating and compensation.

### Animal experiments

All the animal experiments were approved by the Animal Experimental Ethics Committee of Jiangsu Institute of Nuclear Medicine (Wuxi, China). H520 or 1703 cells (5 × 10⁷ cells in 100 μL PBS) were mixed 1:1 (v/v) with Matrigel (Corning, USA) on ice to enhance tumor establishment. The cell suspension was subcutaneously injected into the right flank of male BALB/c nude mice (4 weeks old, 18–20 g) under brief anesthesia with 2% isoflurane in oxygen. Tumor growth was monitored until the average tumor volume reached approximately 100 mm³, after which the mice were randomly assigned into four groups (*n* = 5, per group): vehicle control, AZD4547 (10 mg/kg), Stattic (5 mg/kg), AZD4547 + Stattic combination, AZD4547 in combination with STAT3 knockdown, Stattic as a single agent, Stattic in combination with RRM2 overexpression. Mice were first ranked according to tumor volume (or body weight), stratified into comparable blocks, and then randomly assigned to treatment groups to ensure balanced baseline tumor burdens across groups. Drugs were administered intraperitoneally (i.p.) once daily for 14 consecutive days, and vehicle consisted of 0.5% methylcellulose with 0.1% Tween-80. Tumor dimensions were measured every other day using digital calipers, and tumor volume was calculated using the formula. Volume = Length × (Width)^2^ /2. Body weight and general health status (e.g., activity, grooming, food intake) were recorded throughout the study to assess systemic toxicity. At the study endpoint, mice were euthanized by CO₂ inhalation, and tumors were excised, weighed, photographed, and processed for histological and immunohistochemical analyses.

### Immunohistochemistry (IHC)

Tumor tissues were fixed in 10% neutral-buffered formalin for 24–48 h, embedded in paraffin, and sectioned at 4 μm thickness. Sections were deparaffinized, rehydrated, and subjected to antigen retrieval (citrate buffer, pH 6.0, 95–100 °C, 15 min), followed by 3% H₂O₂ treatment to quench endogenous peroxidase activity. After blocking with 5% BSA, sections were incubated overnight at 4 °C with primary antibodies against Ki67, or RRM2, followed by incubation with HRP-conjugated secondary antibodies. Immunoreactivity was visualized using DAB substrate and counterstained with hematoxylin. Images were acquired using an Olympus IX53 microscope, and positive staining was evaluated in at least five randomly selected high-power fields (HPFs) per section using Image J software for semi-quantitative analysis.

### Serum biochemical analysis in mice

Mice were randomly assigned to different treatment groups. At the experimental endpoint, whole blood samples were collected via retro-orbital venous plexus puncture. The collected blood was allowed to stand at 4 °C and subsequently centrifuged at 4000 rpm for 15 min to obtain serum. The supernatant serum was carefully collected and used for subsequent biochemical analyses.

Serum levels of aspartate aminotransferase (AST) (Rayto, S03040, China) and alanine aminotransferase (ALT) (Rayto, S03030, China) were measured using an automated biochemical analyzer according to the manufacturer’s instructions to evaluate liver function. In addition, blood urea nitrogen (BUN) (Rayto, S03036, China) and creatinine (CR) (Rayto, S03076, China) levels were quantitatively determined to assess renal function.

### Statistical analysis

Data are presented as mean ± SD from at least three independent experiments. Comparisons between two groups were performed using Student’s *t* test, while multiple-group comparisons were analyzed by one-way ANOVA followed by Tukey’s post hoc test (GraphPad Prism 8.0). A *p*-value < 0.05 was considered statistically significant.

## Results

### Activation of IL-6/STAT3 signaling impairs AZD4547’s cytotoxicity in lung squamous cell carcinoma cells

**Fig. 1:**
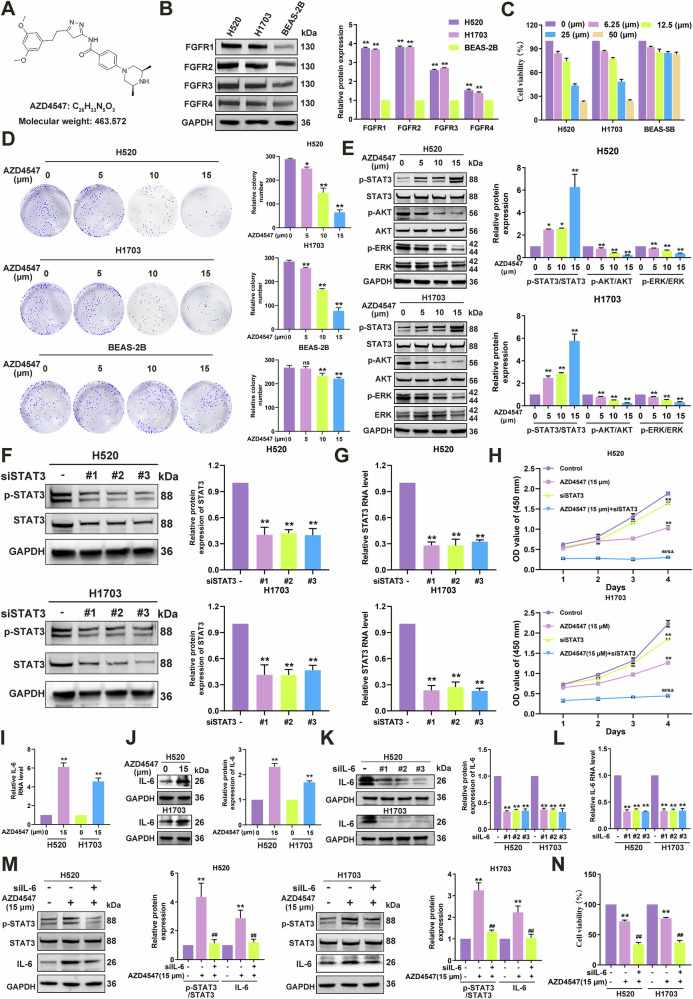
AZD4547 inhibits FGFR signaling but induces compensatory activation of the IL-6/STAT3 axis in lung squamous cell carcinoma cells. **A** Schematic illustration showing that AZD4547 functions as a selective FGFR inhibitor. **B** FGFR expression levels in lung squamous cell carcinoma cell lines (H520 and H1703) and the non-malignant bronchial epithelial cell line BEAS-2B, based on data from the Human Protein Atlas. **C**, **D** MTT and colony-formation assays showing that AZD4547 suppresses the proliferation of H520 and H1703 cells in a dose-dependent manner, whereas BEAS-2B cells exhibit relative resistance to AZD4547 treatment. **E** Western blot analysis of STAT3 phosphorylation and PI3K/AKT and MEK/ERK pathway activity following AZD4547 treatment. **F**, **G** Validation of STAT3 knockdown efficiency in lung squamous cell carcinoma cells by Western blotting and quantitative PCR. **H** Effects of STAT3 knockdown on AZD4547-mediated growth inhibition in lung squamous cell carcinoma cells. **I**, **J** Western blotting and quantitative PCR analyses showing upregulation of IL-6 expression upon AZD4547 treatment. **K**–**M** Effects of IL-6 knockdown on STAT3 phosphorylation, as determined by Western blotting and quantitative PCR. **N** Effects of IL-6 knockdown on the cytotoxic activity of AZD4547 in lung squamous cell carcinoma cells. The results were presented as the Means ± standard deviation (SD), with *n* = 3. *p < 0.05, **p < 0.01 vs. the control group; #*p* < 0.05, ##*p* < 0.01 vs. The AZD4547-treated group; &*p* < 0.05, &*p* < 0.01 vs. the Stattic- knockdown group.

As shown in Fig. 1A, AZD4547 functions as a selective inhibitor of FGFR [26]. Interrogation of the Human Protein Atlas revealed high FGFR expression in the lung squamous cell carcinoma lines H520 and H1703 (Supplementary Figs. S4 and Fig. 1B), whereas the non-malignant human bronchial epithelial line BEAS-2B exhibited low FGFR expression and was used as a control. MTT and colony-formation assays showed that AZD4547 suppressed proliferation of H520 and H1703 cells in a dose-dependent manner, with IC₅₀ values of 21.86 μm and 24.34 μm, respectively; the IC₅₀ for BEAS-2B cells exceeded 100 μm (Fig. 1C, D). Mechanistically, AZD4547 markedly increased STAT3 phosphorylation while concomitantly downregulating the PI3K/AKT and MEK/ERK pathways (Fig. 1E). Accumulating evidence suggests that activation of the STAT3 bypass pathway contributes to resistance to various tyrosine kinase inhibitors (TKIs). Accordingly, STAT3 knockdown lung squamous cell carcinoma cell lines were generated, and effective silencing of STAT3 was validated by Western blotting and quantitative PCR (Fig. 1F, G). Importantly, depletion of STAT3 significantly potentiated the growth-inhibitory effects of AZD4547 in lung squamous cell carcinoma cells (Fig. 1H). Because aberrant IL-6/STAT3 signaling is a recognized driver of tumor growth, survival, invasion, and metastasis [27], we asked whether AZD4547-induced STAT3 activation was mediated by IL-6 upregulation. Western blotting and PCR confirmed a robust increase in IL-6 following AZD4547 treatment (Fig. 1I, J), and IL-6 knockdown substantially reduced STAT3 phosphorylation (Fig. 1K–M). Meanwhile, IL-6 knockdown similarly and significantly enhanced the cytotoxic effect of AZD4547 on lung squamous cell carcinoma cells (Fig. 1N). Collectively, these data indicate that while AZD4547 effectively targets FGFRs, it may paradoxically trigger a compensatory IL-6/STAT3 survival axis, potentially limiting its therapeutic efficacy in FGFR-positive lung squamous cell carcinoma cells.

### AZD4547 induces IL-6/STAT3 activation in a FGFR1-dependent manner in lung squamous cell carcinoma cells

**Fig. 2:**
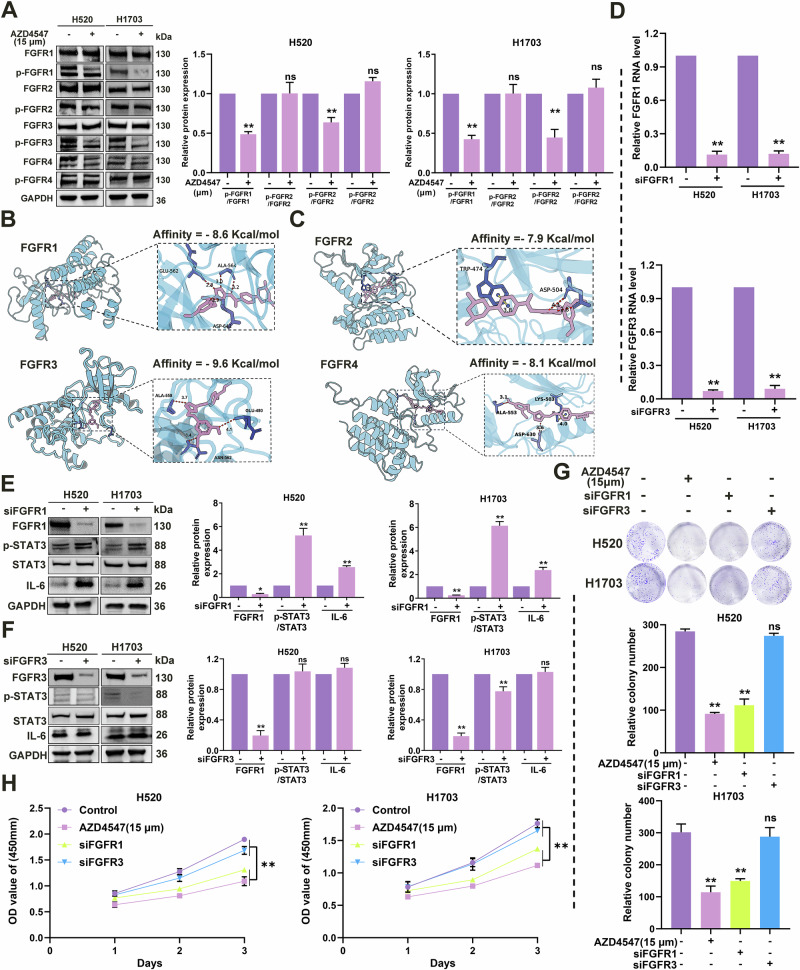
AZD4547 induces IL-6/STAT3 activation in a FGFR1-dependent manner in lung squamous cell carcinoma cells. **A** The protein expression and densitometry analysis of FGFRs and p-FGFRs was assessed in H520 and H1703 cells upon AZD4547 treatment for 24 h. **B**, **C** The molecular docking between AZD4547 and FGFR1-4 was assessed by Autodock vina. **D**–**F** Western blot analysis was performed to analyze the effect of FGFR1 and FGFR3 knockdown on the expression of IL6 and p-STAT3/STAT3 in H520 and H1703 cells. **G** The effects of AZD4547 (15 μm, 7 days) with FGFR1 or FGFR3 knockdown on H520 and H1703 cells were evaluated by colony formation assays. **H** The effects of FGFR1 or FGFR3 knockdown (0–3 days) on the cell viability of H520 and H1703 cells were evaluated by MTT assay. The results were presented as the Means ± standard deviation (SD), with *n* = 3. *p < 0.05; **p < 0.01 *vs*. the control group.

To define how AZD4547 exerts its antitumor activity, we first assessed FGFR protein abundance after treatment. Total FGFR levels in H520 and H1703 cells were unchanged (Fig. 2A), whereas phosphorylation of FGFR1 and FGFR3 was markedly reduced (Fig. 2A). Molecular docking predicted stronger binding of AZD4547 to FGFR1 and FGFR3 (−8.6 and −9.6 kcal/mol; Fig. 2B) than to FGFR2 and FGFR4 (−7.9 and −8.1 kcal/mol; Fig. 2C), consistent with direct engagement of FGFR1/3 and inhibition of their activation. To delineate the contributions of FGFR1/3 to downstream signaling, we generated FGFR1- and FGFR3-knockdown cell lines (Fig. 2D). FGFR1 loss robustly increased IL-6 expression and STAT3 phosphorylation (Fig. 2E), whereas FGFR3 loss had no effect on IL-6 and even reduced STAT3 phosphorylation (Fig. 2F). In viability assays, FGFR1 knockdown suppressed proliferation of both H520 and H1703 cells to an extent comparable to AZD4547 treatment, whereas FGFR3 knockdown had minimal impact despite the drug’s ability to reduce FGFR3 phosphorylation (Fig. 2G, H). Collectively, these data indicate that the antitumor effects of AZD4547 in H520 and H1703 cells are driven primarily by FGFR1 inhibition, which may simultaneously provoke compensatory activation of the IL-6/STAT3 pathway.

### IL-6/STAT3 activation induces RRM2-dependent DNA repair in lung squamous cell carcinoma cells

**Fig. 3:**
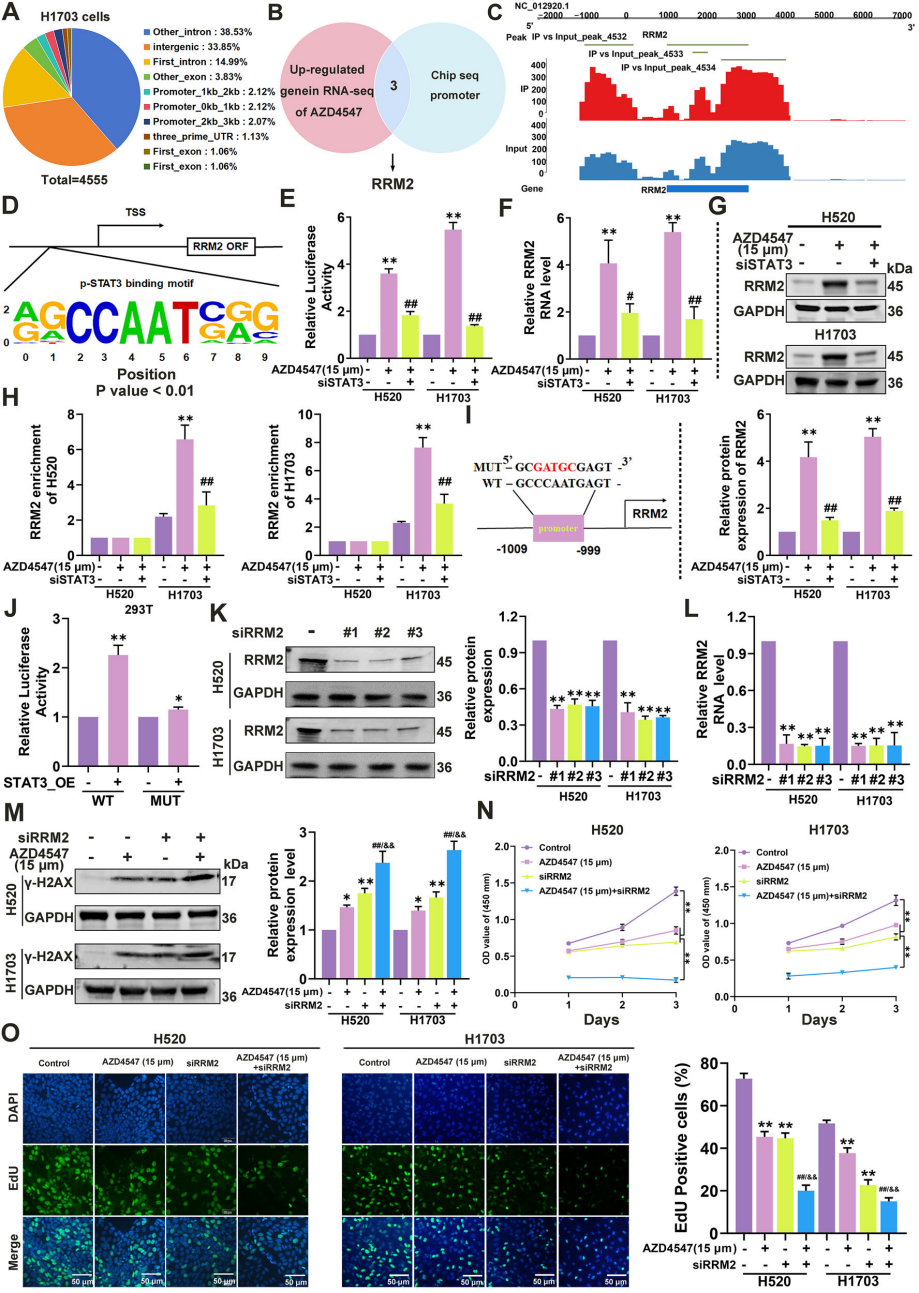
STAT3 directly transcriptionally activates RRM2 to promote DNA repair and attenuate the antitumor efficacy of AZD4547 in lung squamous cell carcinoma cells. **A** Schematic overview of the experimental strategy for identifying STAT3 downstream target genes by ChIP-seq in AZD4547-treated H1703 cells. **B** Venn diagram showing the overlap between genes identified by RNA-seq and STAT3 ChIP-seq analyses. **C** ChIP-seq tracks showing a prominent STAT3-binding peak at the RRM2 promoter region. **D**, **E** Dual-luciferase reporter assays showing increased STAT3-dependent activation of the RRM2 promoter following AZD4547 treatment. **F**, **G** Western blotting and quantitative PCR analyses showing that STAT3 knockdown partially reverses AZD4547-induced upregulation of RRM2 in H520 and H1703 cells. **H** ChIP-qPCR analysis confirming significant enrichment of STAT3 at the RRM2 promoter upon AZD4547 treatment, which is partially abolished by STAT3 knockdown. **I** Schematic representation of luciferase reporter constructs containing the wild-type (WT) or mutant (MUT) STAT3-binding site within the RRM2 promoter. **J** Luciferase reporter assays in 293 T cells showing that STAT3 overexpression significantly enhances WT RRM2 promoter activity but has no effect on the MUT construct. **K**, **L** Validation of RRM2 knockdown efficiency in H520 and H1703 cells by Western blotting and quantitative PCR. **M**, **O** Assessment of DNA damage and DNA synthesis following RRM2 knockdown, as indicated by increased γ-H2AX levels and reduced EdU incorporation. **N** Effects of RRM2 knockdown on AZD4547-induced cytotoxicity in lung squamous cell carcinoma cells. The results were presented as the Means ± standard deviation (SD), with *n* = 3. *p < 0.05, **p < 0.01 vs. the control group; #*p* < 0.05, ##*p* < 0.01 vs. The AZD4547-treated group; &*p* < 0.05, &*p* < 0.01 vs. the RRM2-knockdown group.

To identify STAT3 downstream targets, we performed ChIP-seq in AZD4547-treated H1703 cells (Fig. 3A). Integration of the RNA-seq and ChIP-seq datasets using a Venn diagram revealed a set of overlapping genes (Fig. 3B), among which RRM2 emerged as a key candidate given its central role in DNA synthesis, repair, and replication and its reported ability to reduce drug sensitivity by promoting DNA repair [25–28]. ChIP-seq showed a prominent STAT3-binding peak at the RRM2 promoter (Fig. 3C), suggesting direct transcriptional regulation. Consistently, dual- luciferase reporter assays demonstrated increased STAT3-dependent activation of the RRM2 promoter upon AZD4547 treatment (Fig. 3D, E). Functional validation showed that STAT3 knockdown partially reversed AZD4547-induced upregulation of RRM2 in both H520 and H1703 cells (Fig. 3F, G). In line with this, ChIP-qPCR confirmed significant enrichment of STAT3 at the RRM2 promoter after AZD4547 treatment, which was partially abrogated by STAT3 knockdown (Fig. 3H). Finally, luciferase reporter plasmids containing the wild-type (WT) or mutant (MUT) RRM2 promoter binding site were constructed (Fig. 3I) and transfected into 293 T cells. Luciferase assays revealed that STAT3 overexpression significantly increased the activity of the WT RRM2 promoter reporter, whereas no significant change was observed in the MUT reporter (Fig. 3J). To evaluate the functional consequences of RRM2 loss, we established H520 and H1703 cells with RRM2 knockdown (Fig. 3K, L). RRM2 depletion impaired DNA repair, evidenced by increased γ-H2AX levels and decreased EdU incorporation (Fig. 3M, O). Meanwhile, knockdown of RRM2 similarly and significantly enhanced the cytotoxic effect of AZD4547 on lung squamous cell carcinoma cells (Fig. 3N). Collectively, these findings indicate that STAT3 directly regulates RRM2 transcription in response to AZD4547 and that the STAT3-RRM2 axis modulates DNA damage repair in FGFR1-positive LUSC cells, potentially limiting the efficacy of AZD4547.

### STAT3 inhibitor Stattic and AZD4547 synergistically promote cell death in lung squamous cell carcinoma cells

**Fig. 4:**
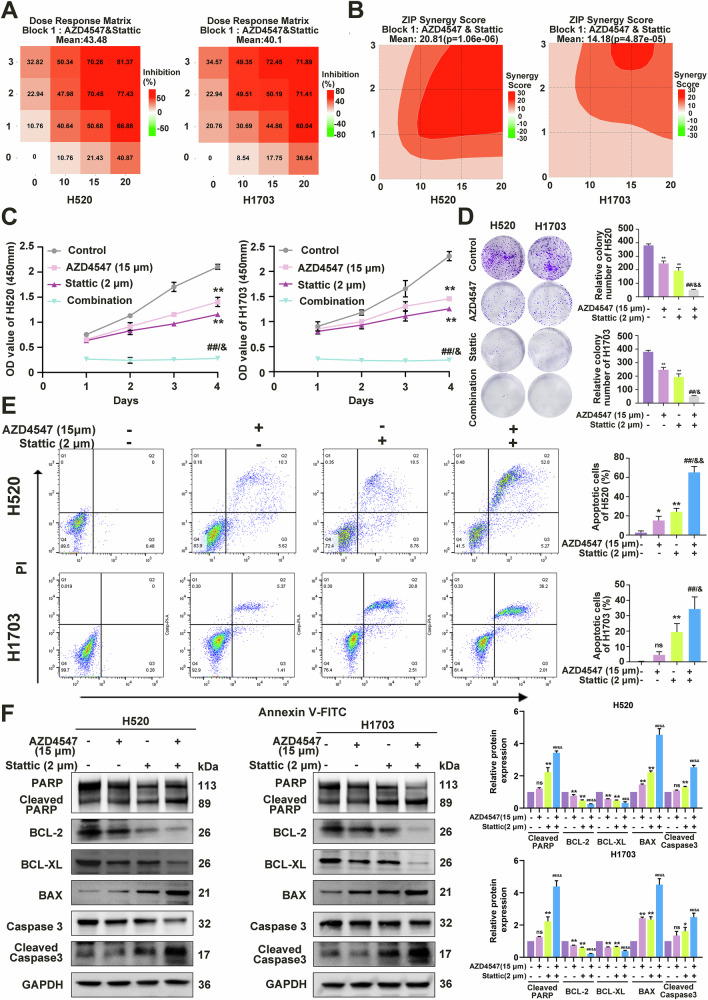
STAT3 inhibitor Stattic and AZD4547 synergistically promote cell death in lung squamous cell carcinoma cells. **A** The relative cell viability of H520 and H1703 cells after 24 h under different doses of AZD4547, Stattic and their combination. **B** Heatmaps of drug combination responses. ZIP Synergy scores were calculated with Synergyfinder software. Scores >0 indicated synergism and scores >10 were considered strongly synergistic. **C** Cell proliferation assays of H1703 and H520 cell lines treated with AZD4547, Stattic, and their combination. Cell viability was measured using an MTT assay at various time points (0–4 days). **D** Colony formation assays showed the effects of the various concentrations of AZD4547 and Stattic on the clonogenic survival of H520 and H1703 cells. **E** Flow cytometric analysis showing that combination treatment resulted in a significantly higher proportion of apoptotic cells compared with either monotherapy. **F** Expression of apoptosis marker proteins after treatment with AZD4547, Stattic, or a combination of H520 and H1703 cells. The results were presented as the Means ± standard deviation (SD), with *n* = 3. *p < 0.05, **p < 0.01 *vs*. the control group; #*p* < 0.05, ##*p* < 0.01 *vs*. The AZD4547-treated group; &*p* < 0.05, &*p* < 0.01 *vs*. the Stattic-treated group.

To test whether blocking IL-6/STAT3 activation could potentiate AZD4547-induced cytotoxicity, we combined the STAT3 inhibitor Stattic with AZD4547 and assessed viability of H520 and H1703 cells. MTT assays across concentration matrices showed a pronounced antitumor effect of the combination (Fig. 4A). Drug-drug interaction analysis using the ZIP model in SynergyFinder yielded synergy scores of 20.81 (H520) and 14.18 (H1703) (Fig. 4B), both above the conventional synergy threshold of 10. At fixed doses (AZD4547, 15 μm; Stattic, 2 μm), the combination produced a marked, time-dependent loss of viability (Fig. 4C). Colony-formation assays likewise demonstrated substantial suppression of clonogenic growth in both lines (Fig. 4D). Both flow cytometric analysis and TUNEL staining demonstrated that combination treatment resulted in a significantly greater proportion of apoptotic cells than either monotherapy (Figs. 4E and Supplementary Fig. S5). Consistently, immunoblotting confirmed enhanced caspase-3 activation-evidenced by cleavage of caspase-3 and its substrate PARP-together with downregulation of the antiapoptotic proteins Bcl-2 and Bcl-xl and upregulation of the proapoptotic protein Bax (Fig. 4F). Collectively, these results demonstrate that AZD4547 and Stattic act synergistically to inhibit proliferation and induce apoptosis in FGFR1-positive lung squamous cell carcinoma cells in vitro, supporting this combination as a promising therapeutic strategy.

### Stattic augments AZD4547’s cytotoxicity by suppressing STAT3-RRM2 in lung squamous cell carcinoma cells

**Fig. 5:**
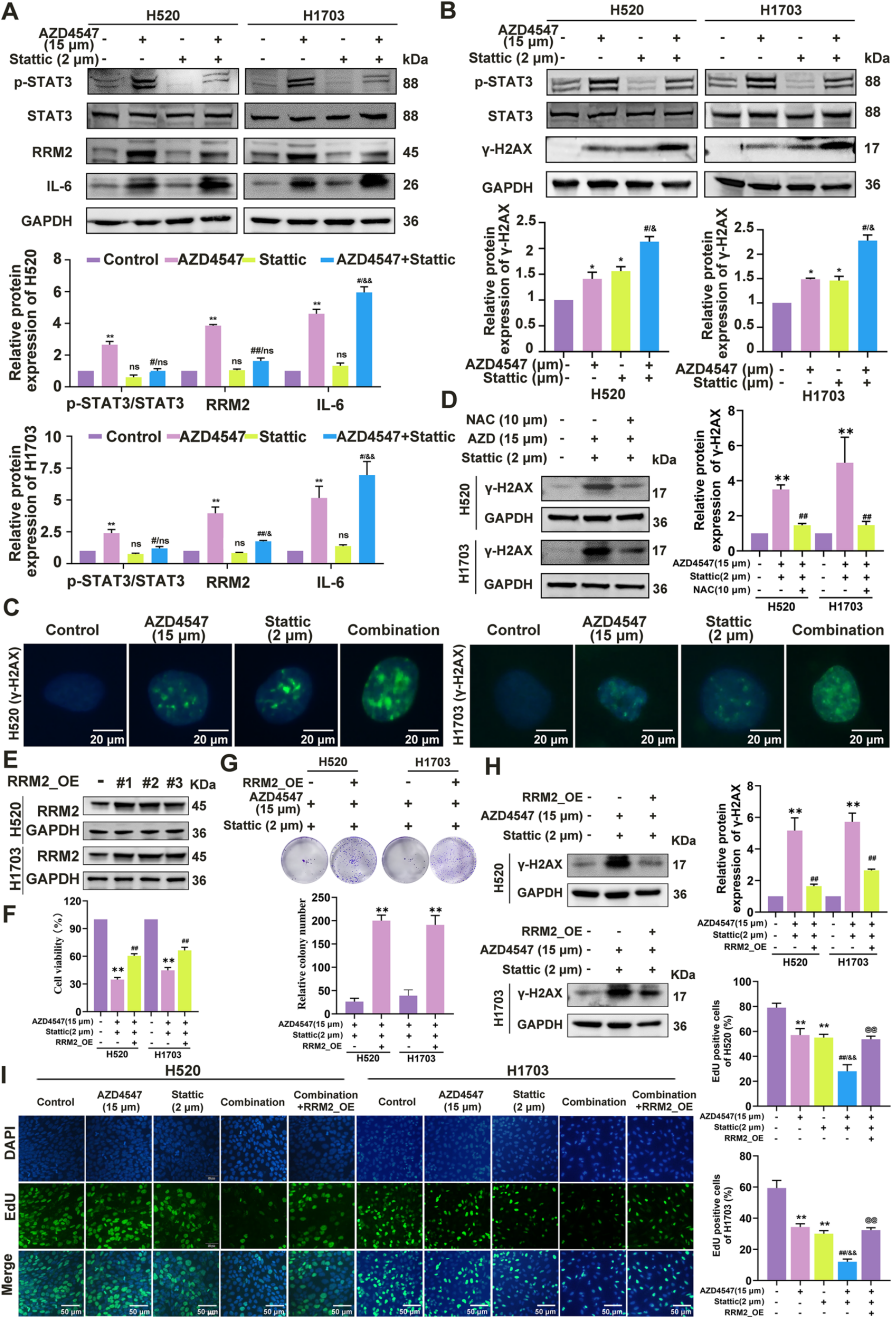
STAT3 inhibitor Stattic augments AZD4547’s cytotoxicity by regulating STAT3-RRM2 signaling in lung squamous cell carcinoma cells. **A**, **B** The expression of RRM2, p-STAT3/STAT3, γ-H2AX and IL-6 were detected by Western blot. **C** Immunofluorescence analysis of γ-H2AX expression. **D** The expression of γ-H2AX, a marker of DNA damage in H520 and H1703 cells. **E** Western blot analysis confirming RRM2 overexpression in the indicated cell lines. **F** MTT assay analysis of the effect of RRM2 overexpression on cytotoxicity of combined drugs. **G** Colony formation assay analysis of the effect of RRM2 overexpression on the cell proliferation ability of combined drugs. **H** Western blot detects expression of γ-H2AX. **I** EdU assay for detecting the DNA synthesis ability of cells. The results were presented as the Means ± standard deviation (SD), with *n* = 3. *p < 0.05, **p < 0.01 *vs*. the control group; #*p* < 0.05, ##*p* < 0.01 *vs*. The AZD4547-treated group or Combination-treated group; &*p* < 0.05, &*p* < 0.01 *vs*. the Stattic-treated group; @@*p* < 0.01 *vs*. Combination-treated group.

To systematically assess how AZD4547 plus Stattic affects the STAT3-RRM2 axis in FGFR1-positive lung squamous cell carcinoma cells, we performed integrated molecular and functional analyses. Combination treatment further increased IL-6 protein levels, indicating compensatory cytokine induction under STAT3 blockade (Fig. 5A). Functionally, co-treatment markedly increased the expression and nuclear accumulation of the double-strand break (DSB) marker γ-H2AX (Fig. 5B, C), while EdU incorporation assays showed a pronounced reduction in DNA synthesis relative to either monotherapy (Fig. 5I). Because excessive reactive oxygen species (ROS) can generate DSBs [28], pretreatment with the antioxidant N-acetyl-L-cysteine (NAC) attenuated the DNA damage induced by the combination (Fig. 5D). To probe the role of RRM2, we established RRM2-overexpressing cell lines (Fig. 5E). RRM2 overexpression partially rescued cell viability (Fig. 5F, G) and DNA-damage repair capacity (Fig. 5H) under combination treatment. Collectively, these results show that AZD4547 and Stattic act synergistically to amplify oxidative DNA damage and suppress the STAT3-RRM2 axis, thereby compromising DNA repair in FGFR1-positive lung squamous cell carcinoma cells.

### Stattic augments AZD4547’s cytotoxicity by inducing ROS-dependent mitochondrial dysfunction in lung squamous cell carcinoma cells

**Fig. 6:**
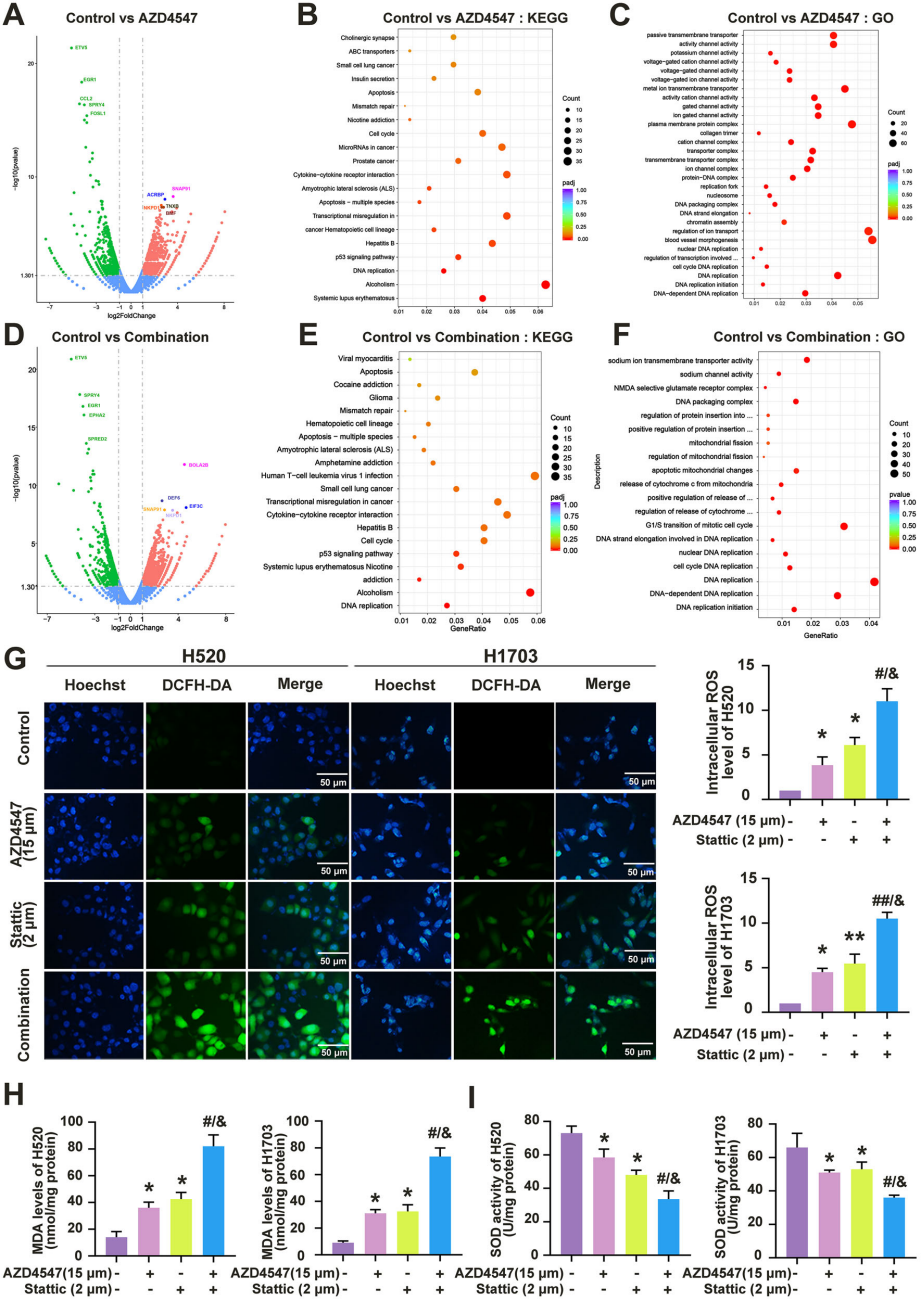
Transcriptomic profiling reveals enhanced oxidative stress induced by combined AZD4547 and Stattic treatment in FGFR1-positive lung squamous cell carcinoma cells. **A** Volcano plot showing differentially expressed genes (DEGs) in H1703 cells treated with AZD4547 versus vehicle control. KEGG pathway enrichment (**B**) and Gene Ontology (GO) enrichment (**C**) analyses of DEGs induced by AZD4547, highlighting pathways involved in DNA replication, cell cycle regulation, mismatch repair, base excision repair, apoptosis, and p53 signaling. **D** Volcano plot showing DEGs in H1703 cells treated with the combination of AZD4547 and Stattic versus vehicle control. KEGG (**E**) and GO (**F**) enrichment analyses of DEGs in the combination group relative to AZD4547 monotherapy, demonstrating significant enrichment in DNA damage repair, cell cycle regulation, apoptosis, and mitochondrial quality control-related processes, including mitophagy, mitochondrial fission, cytochrome c release, and regulation of mitochondrial membrane proteins. **G**–**I** Intracellular oxidative stress markers in H1703 cells following the indicated treatments. Reactive oxygen species (ROS) levels (**G**) and malondialdehyde (MDA) levels (**H**) were increased, whereas superoxide dismutase (SOD) activity (**I**) was decreased after AZD4547 or Stattic treatment alone; these effects were markedly potentiated by the combination. The results were presented as the Means ± standard deviation (SD), with *n* = 3. *p < 0.05, **p < 0.01 *vs*. the control group; #*p* < 0.05, ##*p* < 0.01 *vs*. The AZD4547-treated group; &*p* < 0.05 *vs*. the Stattic-treated group.

**Fig. 7:**
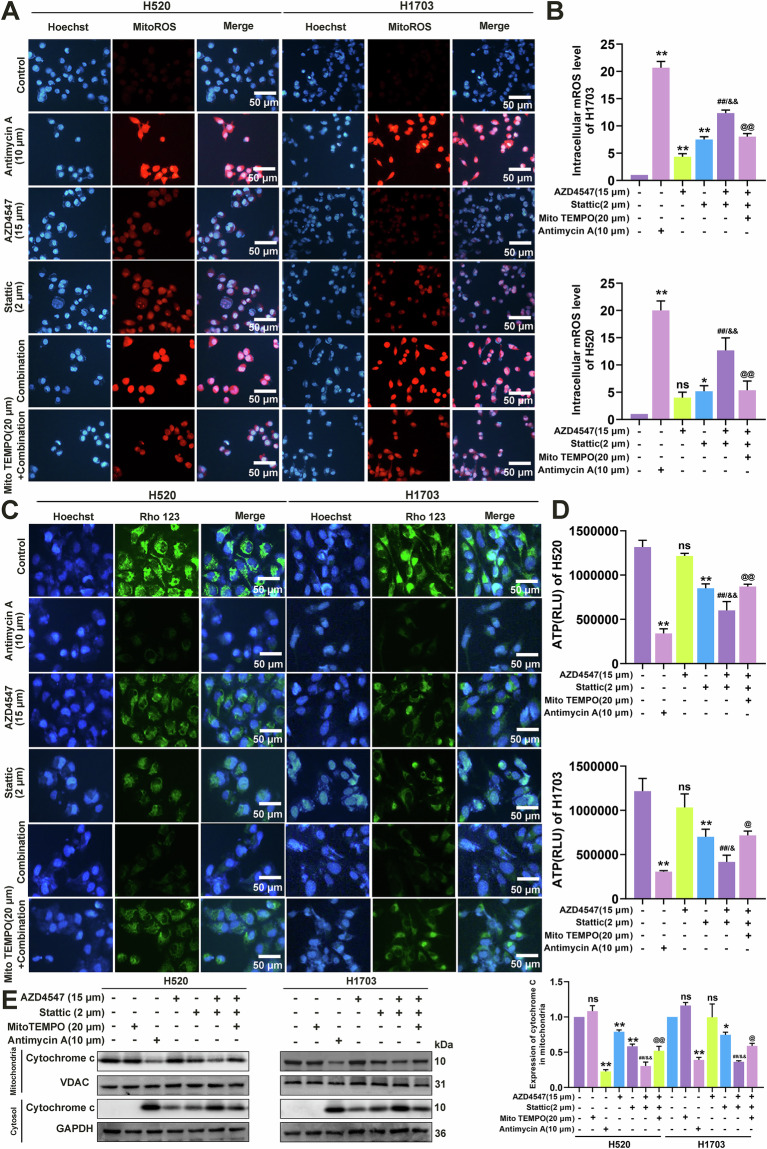
Combined AZD4547 and Stattic treatment triggers mitochondrial ROS accumulation, mitochondrial dysfunction, and cytochrome c-mediated apoptotic signaling in H1703 cells. Representative images (**A**) and quantification (**B**) of mitochondrial superoxide levels detected by MitoSOX Red in H1703 cells following the indicated treatments. Antimycin A was used as a positive control to induce mitochondrial ROS accumulation, whereas MitoTEMPO was used as a mitochondria-targeted ROS scavenger. **C** Mitochondrial membrane potential (ΔΨm) assessed by Rhodamine 123 staining, showing a pronounced loss of ΔΨm in the combination group. **D** Intracellular ATP levels measured after the indicated treatments, demonstrating reduced ATP production upon combination treatment. **E** Immunoblot analysis of cytochrome c in mitochondrial and cytosolic fractions, showing enhanced cytochrome c release from mitochondria to cytosol following combined AZD4547 and Stattic treatment compared with either agent alone; this effect was partially reversed by MitoTEMPO. Together, these results indicate that AZD4547 and Stattic synergistically amplify oxidative stress, induce mitochondrial dysfunction, promote cytochrome c release, and activate apoptotic signaling in FGFR1-positive lung squamous cell carcinoma cells. The results were presented as the Means ± standard deviation (SD), with *n* = 3. * *p* < 0.05; ** *p* < 0.01 *vs*. the control group; # *p* < 0.05, ## *p* < 0.01 *vs*. The AZD4547-treated group or Combination-treated group; & *p* < 0.05, & *p* < 0.01 *vs*. the Stattic-treated group; @ *p* < 0.05, @@ *p* < 0.01 *vs*. Combination-treated group.

To further elucidate how the combination of AZD4547 and Stattic promotes oxidative DNA damage in FGFR1-positive lung squamous cell carcinoma cells, we performed transcriptomic profiling of H1703 cells subjected to vehicle control, AZD4547 or Stattic monotherapy (Supplementary Fig. S6), or combination treatment. Our subsequent analyses focused primarily on the AZD4547-treated and combination-treated groups. Compared with untreated control cells, AZD4547 treatment resulted in 2455 differentially expressed genes (DEGs), including 1479 upregulated and 976 downregulated genes (Fig. 6A). KEGG pathway and Gene Ontology (GO) enrichment analyses revealed that these DEGs were predominantly associated with DNA replication, cell cycle regulation, mismatch repair, base excision repair, apoptosis, and the p53 signaling pathway (Fig. 6B, C). In the combination treatment group, a total of 2498 genes were differentially expressed, with 1587 genes upregulated and 911 genes downregulated relative to the control group (Fig. 6D). Notably, compared with AZD4547 treatment alone, the differentially expressed genes in the combination group were not only significantly enriched in pathways related to DNA damage repair, cell cycle regulation, and apoptosis, but also showed pronounced enrichment in mitochondrial quality control and apoptotic processes, including mitophagy, mitochondrial fission, cytochrome c release, and regulation of mitochondrial membrane proteins (Fig. 6E, F). Given the tight link between oxidative stress and mitochondrial dysfunction, we next quantified intracellular oxidative-stress markers. Either AZD4547 or Stattic alone increased ROS and malondialdehyde (MDA) levels while decreasing superoxide dismutase (SOD) activity, whereas the combination produced substantially larger changes (Fig. 6G–I), indicative of heightened oxidative stress. Using MitoSOX Red, we next assessed mitochondrial reactive oxygen species (mtROS) levels in the combination treatment group. Antimycin A, which inhibits mitochondrial respiratory chain complex III and disrupts electron transport, thereby causing excessive accumulation of mitochondrial ROS and subsequent collapse of mitochondrial membrane potential, was used as a positive control. In contrast, MitoTEMPO, a mitochondria-targeted antioxidant, was employed to scavenge mitochondrial ROS. Latter, we detected elevated mitochondrial superoxide in the combination group, consistent with impaired mitochondrial ROS clearance (Fig. 7A, B). At the same time, Rhodamine 123 staining revealed a marked loss of mitochondrial membrane potential (Fig. 7C), accompanied by a significant reduction in ATP production (Fig. 7D). Finally, subcellular fractionation showed enhanced release of cytochrome c from mitochondria to cytosol with the combination versus either agent alone (Fig. 7E), consistent with mitochondrial damage and apoptotic activation. However, the effects induced by the combination treatment were partially reversed by MitoTEMPO. Together, these data demonstrate that AZD4547 and Stattic synergistically amplify oxidative stress, drive mitochondrial dysfunction, promote cytochrome-c release, and activate caspase-3-mediated apoptosis in FGFR1-positive lung squamous cell carcinoma cells.

### STAT3 inhibitor Stattic and AZD4547 synergistically promote cell death in xenografted models

**Fig. 8:**
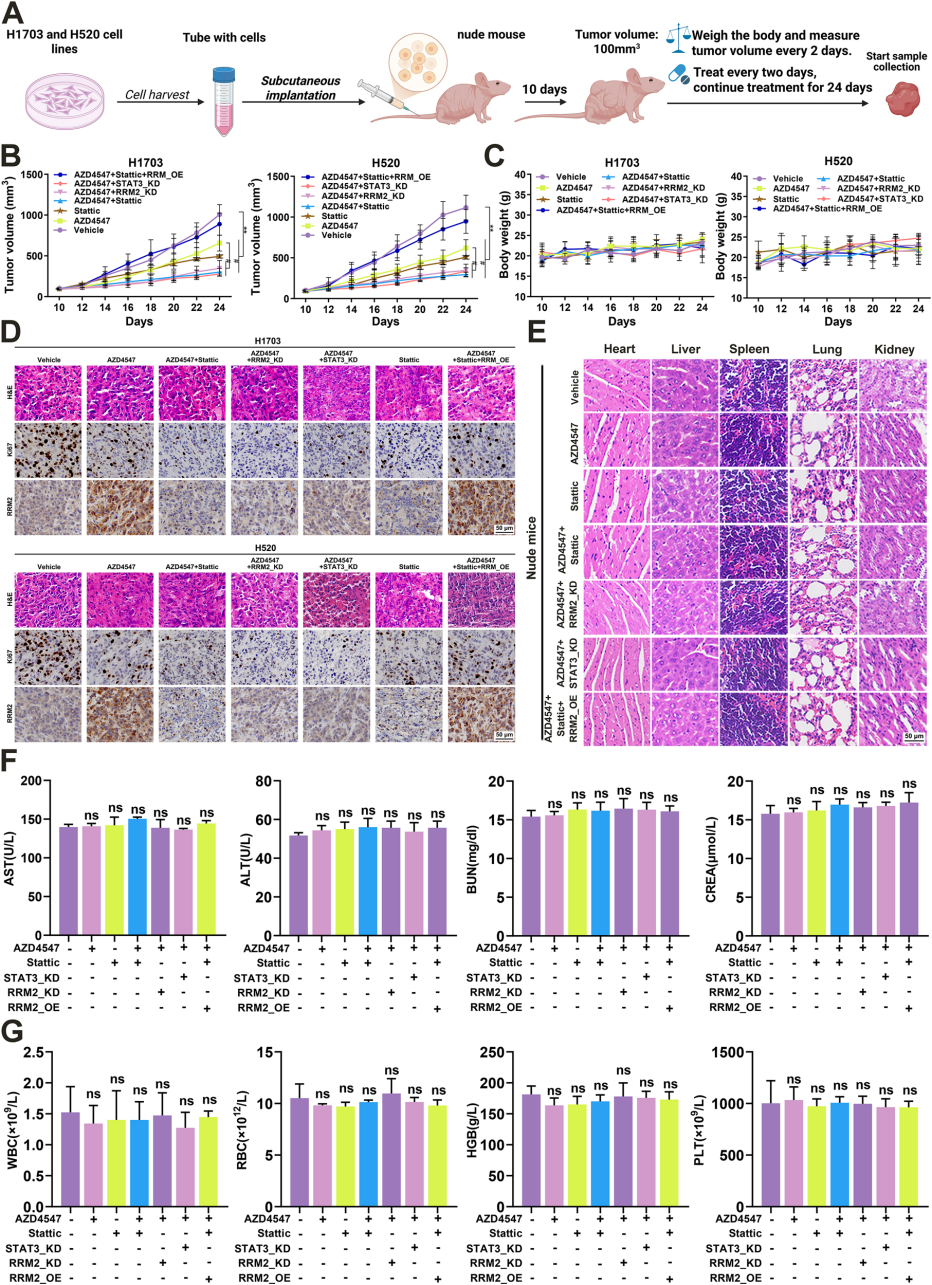
Stattic synergizes with AZD4547 to suppress tumor growth in FGFR1-positive lung squamous cell carcinoma xenografts with favorable tolerability. **A** Schematic of the in vivo xenograft study design and treatment regimens. H520 and H1703 tumor-bearing mice (tumor volume ~100 mm³) were randomized to receive vehicle (PBS), AZD4547 (10 mg/kg), Stattic (5 mg/kg), AZD4547 + Stattic (10 mg/kg + 5 mg/kg), or the indicated genetic manipulation groups (RRM2 knockdown [RRM2_KD], STAT3 knockdown [STAT3_KD], and RRM2 overexpression [RRM2_OE]) with the corresponding drug treatments. Tumor volume and body weight were monitored every 2 days. **B** Tumor growth curves of H520 and H1703 xenografts under the indicated treatments. AZD4547 significantly inhibited tumor growth compared with the vehicle group, and its antitumor efficacy was further enhanced by Stattic co-treatment as well as in RRM2_KD and STAT3_KD tumors. In contrast, RRM2 overexpression markedly attenuated the antitumor activity of the AZD4547-Stattic combination. **C** Body weight changes during treatment, showing no significant differences among groups. **D** Representative H&E staining and immunohistochemical (IHC) staining for Ki-67 and RRM2 in tumor tissues. Compared with AZD4547 monotherapy, the combination treatment markedly reduced Ki-67 and RRM2 expression, whereas RRM2 overexpression significantly reversed these effects. **E** Representative H&E staining of major organs (heart, liver, spleen, lung, and kidney) from each group, showing no overt histopathological abnormalities. **F** Serum biochemistry analysis of liver and kidney function markers (AST, ALT, BUN, and creatinine). Although mild elevations were observed in the monotherapy and combination groups-most notably in the combination group-all parameters remained within the normal range, indicating no apparent hepatic or renal dysfunction. **G** The CBC values of animals in each group were maintained within the normal fluctuation range of the vehicle group. Furthermore, the white blood cell counts in all groups were at a low level, which was consistent with the immunodeficient phenotype of the mice, indicating that the experimental model was in a stable state. The results were presented as the Means ± standard deviation (SD), with *n* = 5. *p < 0.05; **p < 0.01 *vs*. the vehicle group; # *p* < 0.05 *vs*. The AZD4547-treated group.

To assess in vivo synergy between AZD4547 and Stattic and to define the contribution of RRM2 to AZD4547 response, we established H520 and H1703 xenografts. When tumors reached ~100 mm³, mice were treated with PBS, AZD4547 alone (10 mg/kg), AZD4547 (10 mg/kg) plus Stattic (5 mg/kg), or xenografts derived from RRM2-knockdown cells (RRM2_KD), AZD4547 in combination with STAT3 knockdown, Stattic as a single agent, Stattic in combination with RRM2 overexpression. Tumor volumes and body weights were recorded every two days (Fig. 8A). As shown in Fig. 8B, AZD4547 significantly suppressed tumor growth compared with the vehicle group, and this antitumor effect was further enhanced by co-treatment with Stattic as well as in RRM2_KD and STAT3_KD tumors. In contrast, RRM2 overexpression markedly attenuated the therapeutic efficacy of the AZD4547-Stattic combination. Histopathological analyses-including H&E staining and IHC for Ki-67 and RRM2-revealed that, compared with AZD4547 monotherapy, the combination regimen substantially decreased Ki-67 and RRM2 expression in tumor tissues, Similarly, this effect was significantly reversed by RRM2 overexpression (RRM2_OE) (Fig. 8D), consistent with our in vitro findings. Importantly, none of the treatment arms produced significant changes in body weight (Fig. 8C) or detectable pathological abnormalities in major organs (Fig. 8E). In addition, serum samples were collected from mice in each group after treatment to evaluate hepatic and renal function. Compared with the vehicle group, the monotherapy and combination groups showed varying degrees of increases in AST, ALT, BUN, and creatinine levels, with the combination group exhibiting the most pronounced elevations. However, AST, ALT, BUN, and creatinine levels in all treatment groups remained within the normal range (Fig. 8F), indicating no overt impairment of liver or kidney function. In addition, no statistically significant differences in the main indicators of complete blood count (CBC) of mice in all dose groups, including white blood cell (WBC) count, red blood cell (RBC) count, hemoglobin (HGB) concentration and platelet (PLT) count, were observed compared with the vehicle group (Fig. 8G). Overall, the combination of AZD4547 and Stattic was well tolerated in mice and demonstrated an acceptable biosafety profile. Together, these in vivo data demonstrate that AZD4547 combined with Stattic robustly inhibits the growth of FGFR1-positive lung squamous cell carcinoma, primarily by suppressing AZD4547-induced activation of the STAT3-RRM2 axis and thereby enhancing antitumor efficacy.

## Discussion

This study proposes a combinatorial therapeutic strategy using the STAT3 inhibitor Stattic in combination with the pan-FGFR inhibitor AZD4547 for FGFR-positive lung squamous cell carcinoma, and elucidates functionally important crosstalk between FGFR and STAT3 signaling. Rather than emphasizing novelty at the level of combination therapy per se, our work defines a distinct mechanistic framework underlying the observed synergy. Specifically, we identify an IL-6/STAT3-RRM2 regulatory axis through which STAT3 directly controls RRM2 transcription, thereby modulating DNA repair capacity. Disruption of this axis, together with FGFR inhibition, exacerbates DNA damage and promotes ROS-driven mitochondrial dysfunction, revealing a cooperative vulnerability that enhances antitumor efficacy. These findings provide mechanistic insight and a conceptual basis for the rational development of targeted combination therapies in this aggressive cancer subtype.

Despite advances in oncology, treatment options for lung squamous cell carcinoma remain limited, and no definitive therapeutic targets have been established. Aberrant activation of fibroblast growth factor receptors (FGFRs) has emerged as a potential therapeutic vulnerability, with FGFR alterations not only driving oncogenic signaling but also correlating with poor prognosis in lung squamous cell carcinoma patients [29]. AZD4547, a selective FGFR1-3 inhibitor, has shown antitumor activity in several cancers [30–33]; however, its efficacy in lung squamous cell carcinoma has been modest [8]. Clinical studies, including the Lung-MAP platform trial, suggest that tumor heterogeneity, co-occurring mutations, and variability within the 8p11 amplicon underlie this limited response [13]. Preclinical evidence indicates that AZD4547 suppresses pro-survival signaling while paradoxically enhancing STAT3 phosphorylation and combining FGFR inhibition with STAT3 blockade yields superior efficacy [34]. In our study, AZD4547 treatment activated IL-6/STAT3 signaling in lung squamous cell carcinoma cell lines, and FGFR1 knockdown exert the same effects, underscoring FGFR1’s role in pathway activation. Importantly, combining AZD4547 with the STAT3 inhibitor Stattic exerted synergistic antitumor effects, supporting this strategy as a promising approach to overcome resistance to FGFR-targeted monotherapy in lung squamous cell carcinoma.

In FGFR2-mutant endometrial cancer, AZD4547 has been shown to induce mitochondrial depolarization, cytochrome c release, and impaired respiration [35]. However, whether a similar vulnerability exists in lung squamous cell carcinoma has remained unclear. Here, we identify a previously unrecognized mechanism whereby dual blockade of FGFR signaling by AZD4547 and STAT3 signaling by Stattic synergistically disrupts DNA damage repair (DDR). DDR is a critical surveillance network that preserves genomic stability, and its dysregulation promotes tumorigenesis while creating exploitable therapeutic vulnerabilities [36]. Transcriptomic profiling revealed that AZD4547 monotherapy reshapes DNA repair-related pathways in lung squamous cell carcinoma cells. Given that STAT3 signaling has been implicated in enhancing DDR capacity, persistent STAT3 activity may attenuate the efficacy of FGFR inhibition [37]. Consistent with this notion, combined treatment with AZD4547 and Stattic markedly increased γ-H2AX accumulation, indicating impaired DNA repair fidelity and highlighting STAT3 as a key mediator of resistance to FGFR-targeted therapy. Mechanistically, our findings further uncover the role of STAT3 downstream target RRM2 in this process. RRM2 is essential for maintaining deoxynucleotide pools required for DNA synthesis and repair, and its paralog RRM2B plays a crucial role in DNA damage repair under oxidative stress conditions [38, 39]. In our experiments, knockdown of RRM2 significantly impaired DNA repair capacity, thereby sensitizing cells to genotoxic stress. Notably, loss of RRM2 was closely associated with accumulation of reactive oxygen species (ROS) and mitochondrial damage [40]. Thus, while STAT3 inhibitors suppress STAT3 activity, they also indirectly downregulate RRM2 transcription, and promotes DNA damage. This, in turn, compromises DNA repair fidelity and synergizes with FGFR inhibition to amplify antitumor effects.

ROS are chemically reactive molecules derived from oxygen, including superoxide anions, hydrogen peroxide, and hydroxyl radicals. Under physiological conditions. ROS function as signaling intermediates that regulate diverse cellular processes. However, excessive ROS can damage DNA, proteins, and lipids, thereby contributing to genomic instability and cell death [28]. In contrast to normal cells, cancer cells are characterized by increased levels of ROS. This elevated ROS drives the activation of numerous pathways essential for cellular transformation and tumor development. Indeed, oxidative stress plays a central role throughout cancer progression, spanning initiation, promotion, and metastasis [41]. Experiments in mouse fibroblasts have demonstrated that elevated ROS levels induce replication stress, thereby promoting chromosomal instability in association with age-related mitochondrial dysfunction [42]. Pharmacological inhibition of STAT3 has been shown to rapidly compromise mitochondrial function, leading to loss of membrane potential and acute ROS generation [43]. In line with this, we observed that dual inhibition with AZD4547 and Stattic substantially elevated intracellular ROS levels and lipid peroxidation, as reflected by increased malondialdehyde (MDA). This oxidative stress established a feed-forward loop of mitochondrial collapse, cytochrome c efflux [44], and apoptotic signaling, further amplifying DNA damage responses [45, 46]. Collectively, these results delineate a dual vulnerability in lung squamous cell carcinoma: combined FGFR and STAT3 inhibition not only destabilizes mitochondrial homeostasis but also disrupts DNA damage repair through RRM2 suppression, thereby intensifying oxidative DNA damage and yielding potent antiproliferative effects. This mechanistic insight provides a strong rationale for pursuing combinatorial therapeutic strategies targeting both FGFR and STAT3 pathways in FGFR1-positive lung squamous cell carcinoma.

This study has several limitations. First, the full spectrum of its regulatory role requires further elucidation. Second, the mechanistic basis of ROS generation during oxidative stress, as well as the distinct contributions of each monotherapy to the synergistic antitumor effects observed, remain to be clarified. Besides, one limitation of this study is the absence of murine pharmacokinetic (PK) analysis; future PK evaluation in mice will be important for fully characterizing systemic exposure and potential drug-drug interactions. These aspects warrant comprehensive exploration in future studies.

## Conclusion

**Fig. 9:**
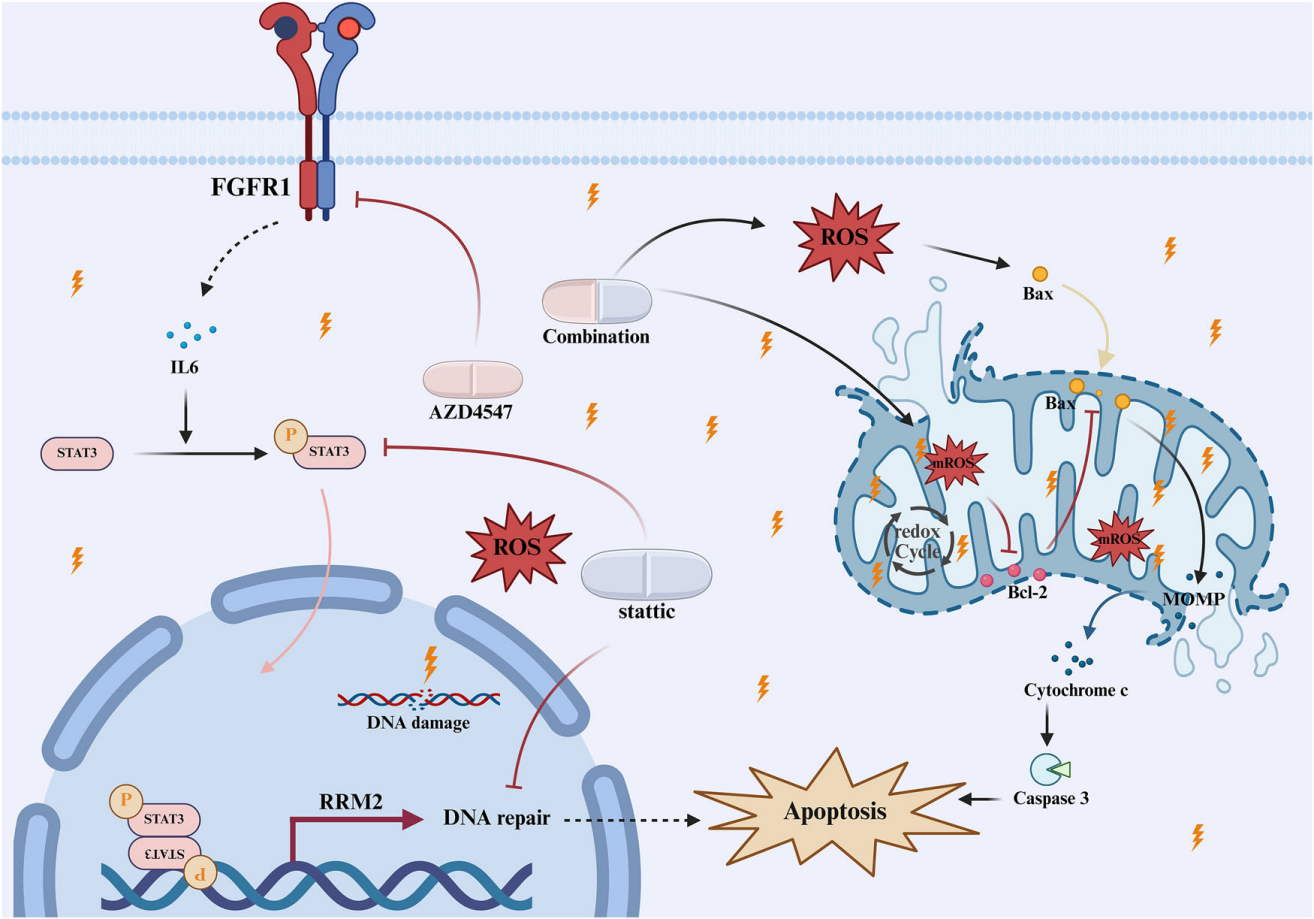
AZD4547 molecular mechanism of combined Stattic against FGFR1-positive lung squamous cell carcinoma cells. (Created with BioGDP.com).

In summary, our study demonstrates that the pan-FGFR inhibitor AZD4547 effectively suppresses FGFR1-positive lung squamous cell carcinoma, while simultaneously triggering compensatory activation of the IL-6/STAT3 signaling pathway (Fig. 9). Notably, combined inhibition of STAT3 with Stattic synergistically enhances the antitumor effects of AZD4547 in vitro and in vivo by inhibiting STAT3-RRM2 activation and thus promoting DNA damage. In addition, Stattic augments AZD4547’s cytotoxicity by inducing ROS-dependent mitochondrial dysfunction (Fig. 9). Overall, these findings reveal a novel interplay between FGFR and STAT3 signaling in lung squamous cell carcinoma and provide a compelling preclinical rationale for dual targeting of FGFR and STAT3 to overcome resistance and improve therapeutic outcomes in FGFR1-positive lung cancers.

## Supplementary information

qPCR original date

Supplementary Figures 1-6

(WB) original data

## Data Availability

The data that support the findings of this study are available from the corresponding author upon reasonable request.
