# Supplementary material for: Stattic enhances the anti-tumor activity of AZD4547 in LUSC by blocking STAT3/RRM2-mediated DNA repair and inducing ROS-driven mitochondrial dysfunction
Source: Cell Death Dis. 2026 May 19;17(1):632. doi: 10.1038/s41419-026-08848-1 (PMC13350979; doi:10.1038/s41419-026-08848-1)
Supplement: Supplementary file 2 — Supplementary Figures 1-6 [file 41419_2026_8848_MOESM2_ESM.docx]

**Supplementary Figure 1**

| **PCR primers** | **Forward (5’-3’)** | **Reverse (5’-3’)** |
| --- | --- | --- |
| STAT3 | CAGCAGCTTGACACACGGTA | AAACACCAAAGTGGCATGTGA |
| IL-6 | GGTGTTGCCTGCTGCCTTCC | GCTCTGGCTTGTTCCTCACTACTC |
| FGFR1 | CCCGTAGCTCCATATTGGACA | TTTGCCATTTTTCAACCAGCG |
| FGFR3 | TGCGTCGTGGAGAGAAGATTT | GCACGGTAACGTAGGGTGTGT |
| RRM2 | GGCTGGCTGTGACTTACCAT | AGCAGTGAGGCTGCATCTTT |
| GAPDH | GGAGGGAGATCCCCCCCCAAAT | GGCTGTGTGTCATACTTCATGG |

All primers used in this study were synthesized by Sangon Biotech (Shanghai) Co., Ltd.

**Supplementary Figure 2**

| **Name** | **siRNA** | **Sense** |
| --- | --- | --- |
| STAT3 | siRNA1 | 5’-CUGCUAAGAUUCAGUGAAATT-3’ |
|  | siRNA2 | 5’-GUGGUGAUCUCCAACAUCUTT-3’ |
|  | siRNA3 | 5’-GGCCAGCAAAGAAUCACAUTT-3’ |
| IL-6 | siRNA1 | 5’-CAGAACAUGUGACATT-3’ |
|  | siRNA2 | 5’-CUUCCAAUCUGGAUUCAAUTT-3’ |
|  | siRNA3 | 5’-GGACAUGACAACUCAUCUCTT-3’ |
| RRM2 | siRNA1 | 5’-GGAGAGUAAAAAUATT-3’ |
|  | siRNA2 | 5’-GAGCCGCUGAGAGAGAAT-3’ |
|  | siRNA3 | 5’-GGAGGAGAGAGAAGAGAATT-3’ |
| FGFR1 | siRNA | 5’-GCAGUGACACCACCUUTT-3’ |
| FGFR3 | siRNA | 5’-GCAUUGGAGGCAUCAAGCUTT-3’ |

All siRNAs used in this study were obtained from GenePharma (Shanghai, China).

**Supplementary Figure 3**

| CHIP-qPCR  (Promoter) | GENE | Forward (5’-3’) | Reverse (5’-3’) |
| --- | --- | --- | --- |
|  | RRM2 | 5’-CATATTTGACGCCCAATGAGTAGT-3’ | 5’-TCCTGGGAGAAAGTTCACAGA-3’ |

All primers used in this study were synthesized by Sangon Biotech (Shanghai) Co., Ltd.

**Supplementary Figure 4**


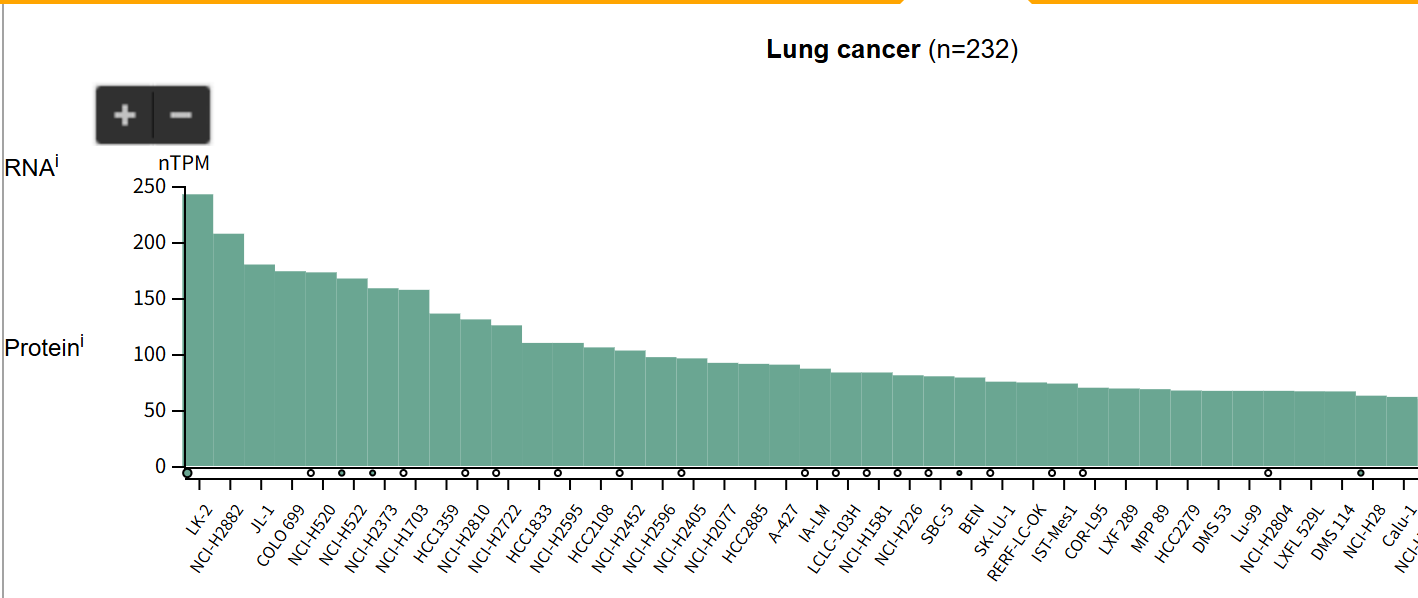


**Figure S4:** The protein expression of FGFR1 in H520 and H1703 cells in The Human Protein Atlas database.

**Supplementary Figure 5**

**
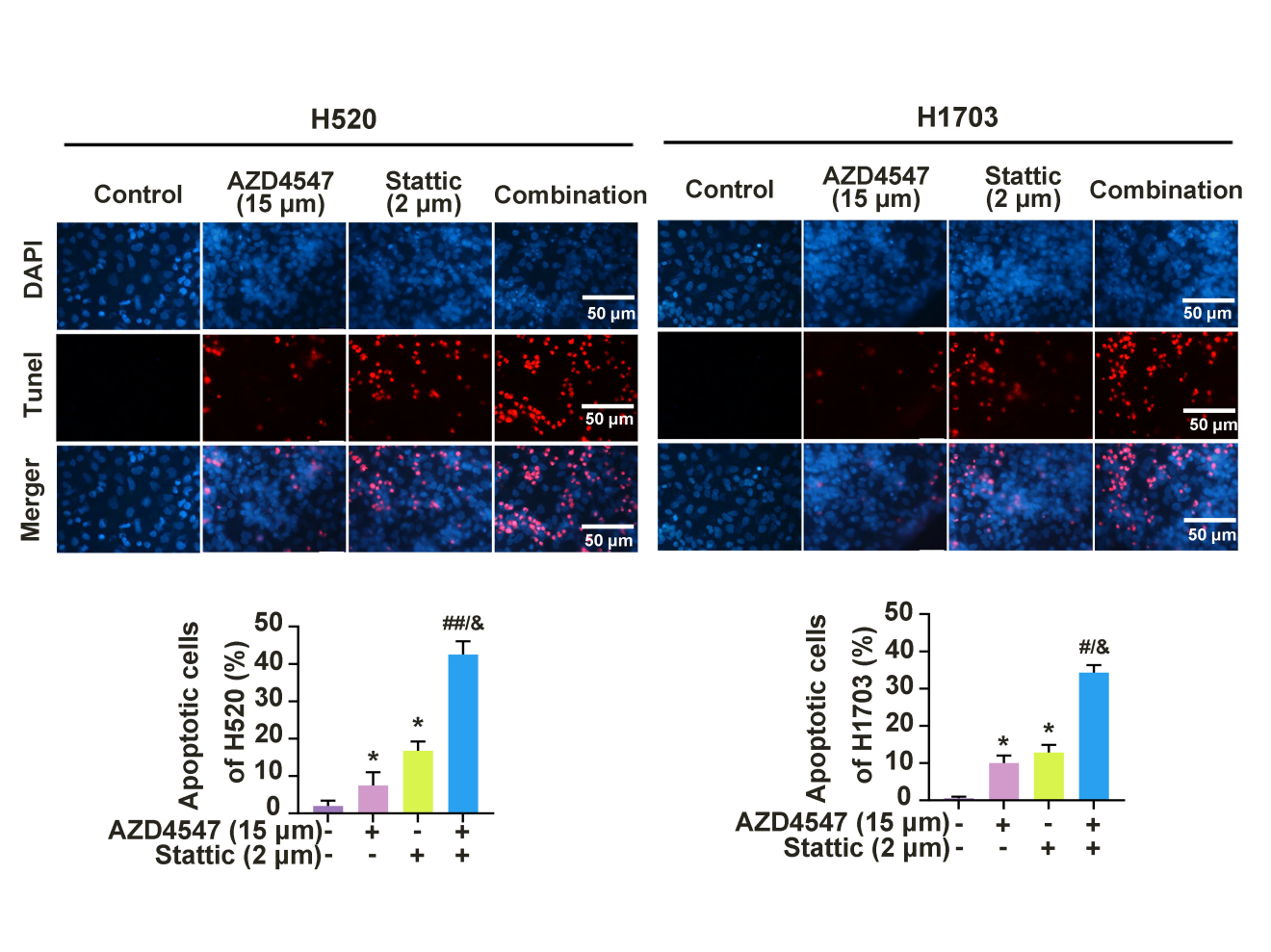
**

**Figure S5:** After exposure to AZD4547, Stattic, or a combination of the two, H520 and H1703 cells underwent apoptosis as detected by TUNEL staining.

**Supplementary Figure 6**

**C**

**
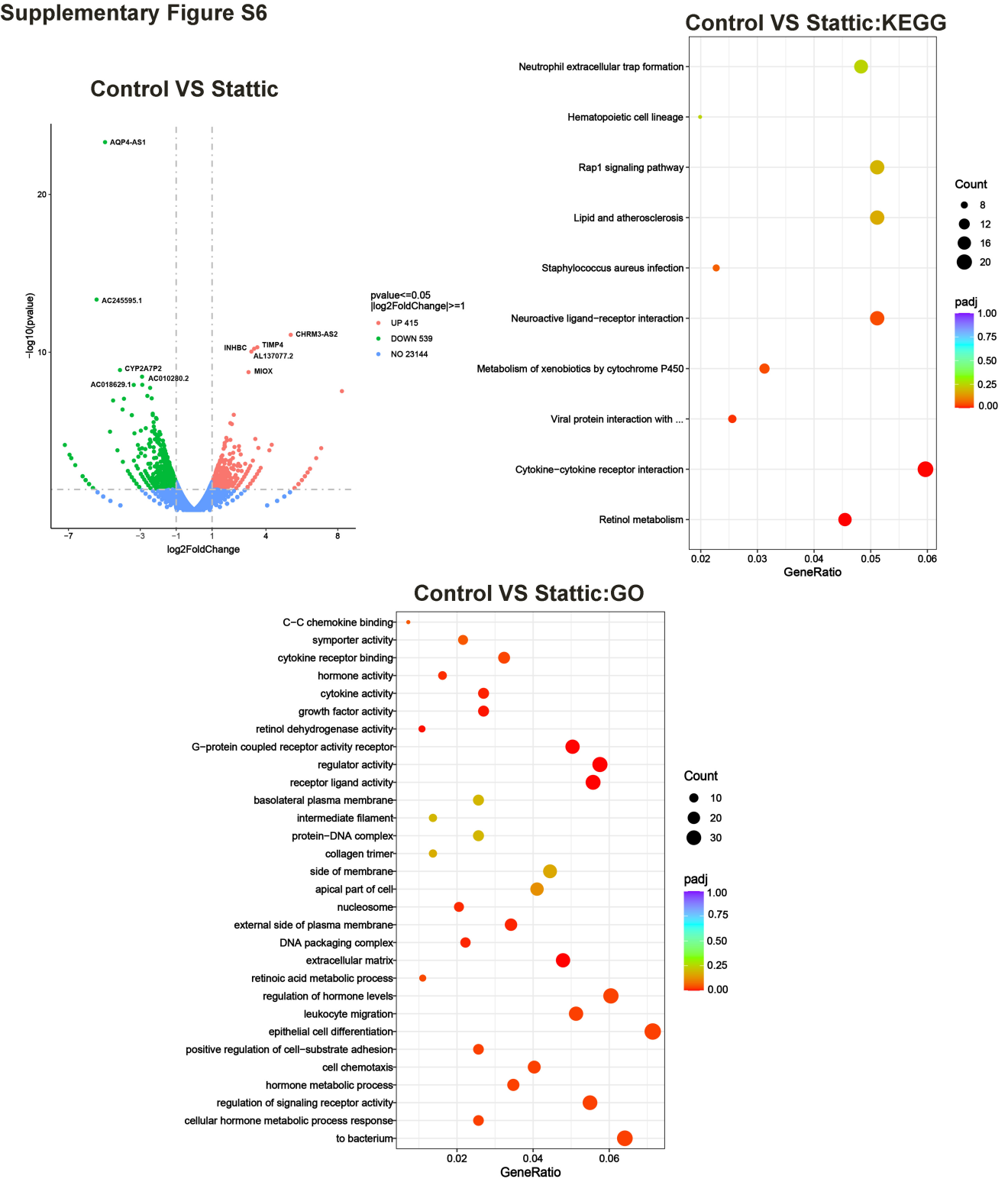
**

**B**

**A**

**A:** Volcano plots of DEGs with log2 fold change ≥ 1 (pvalue ≤ 0.05). **B:** Gene ontology analysis of DEGs. **C:** Kyoto Encyclopedia of Genes and Genomes analysis of DEGs
