# Supplementary material for: Stattic enhances the anti-tumor activity of AZD4547 in LUSC by blocking STAT3/RRM2-mediated DNA repair and inducing ROS-driven mitochondrial dysfunction
Source: Cell Death Dis. 2026 May 19;17(1):632. doi: 10.1038/s41419-026-08848-1 (PMC13350979; doi:10.1038/s41419-026-08848-1)
Supplement: Supplementary file 3 — (WB) original data [file 41419_2026_8848_MOESM3_ESM.pdf]

Uncropped gels for Westem Blots in Figure 1

F1-B

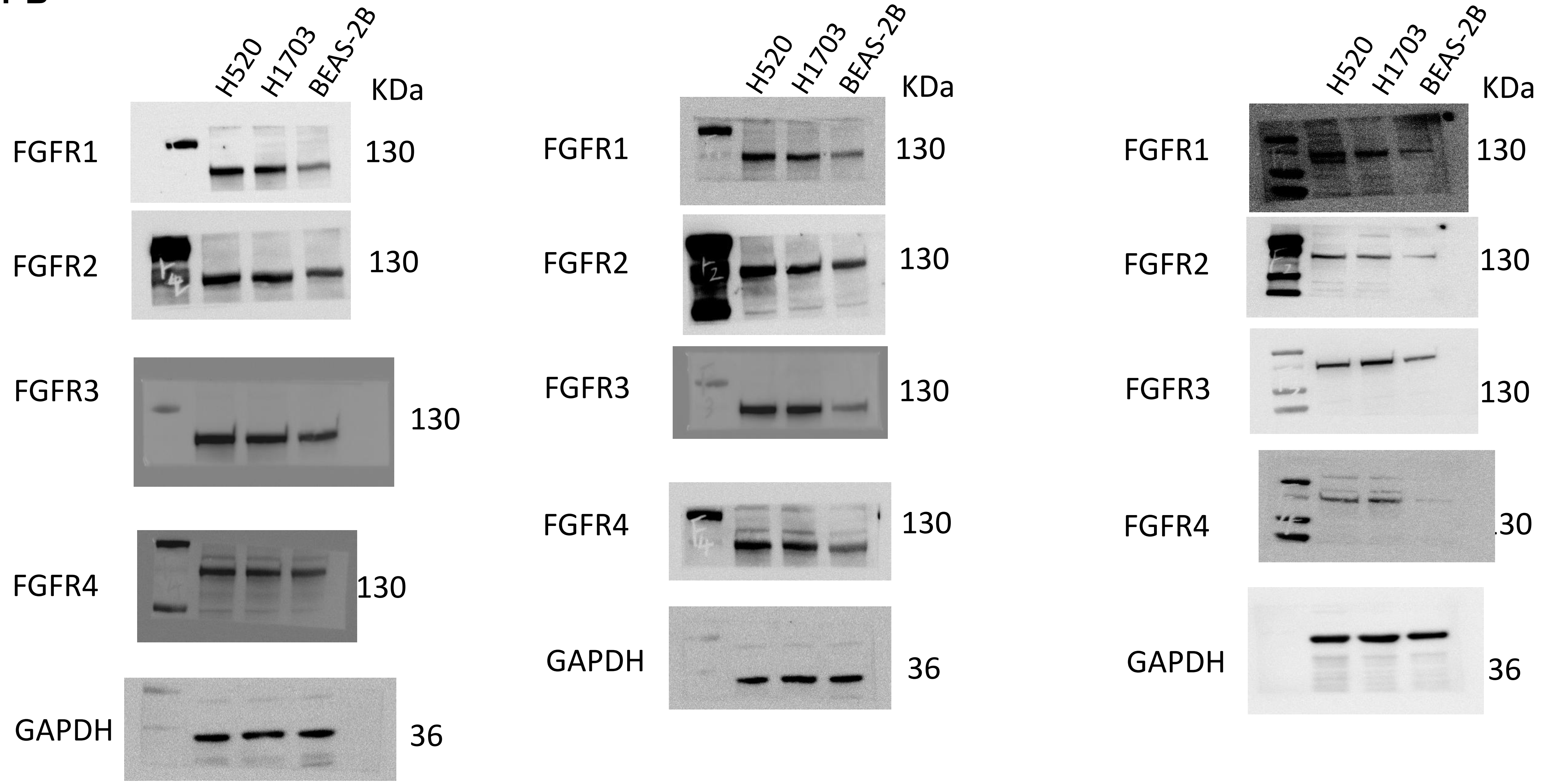

Uncropped gels for Westem Blots in Figure 1

F1-E(H520)

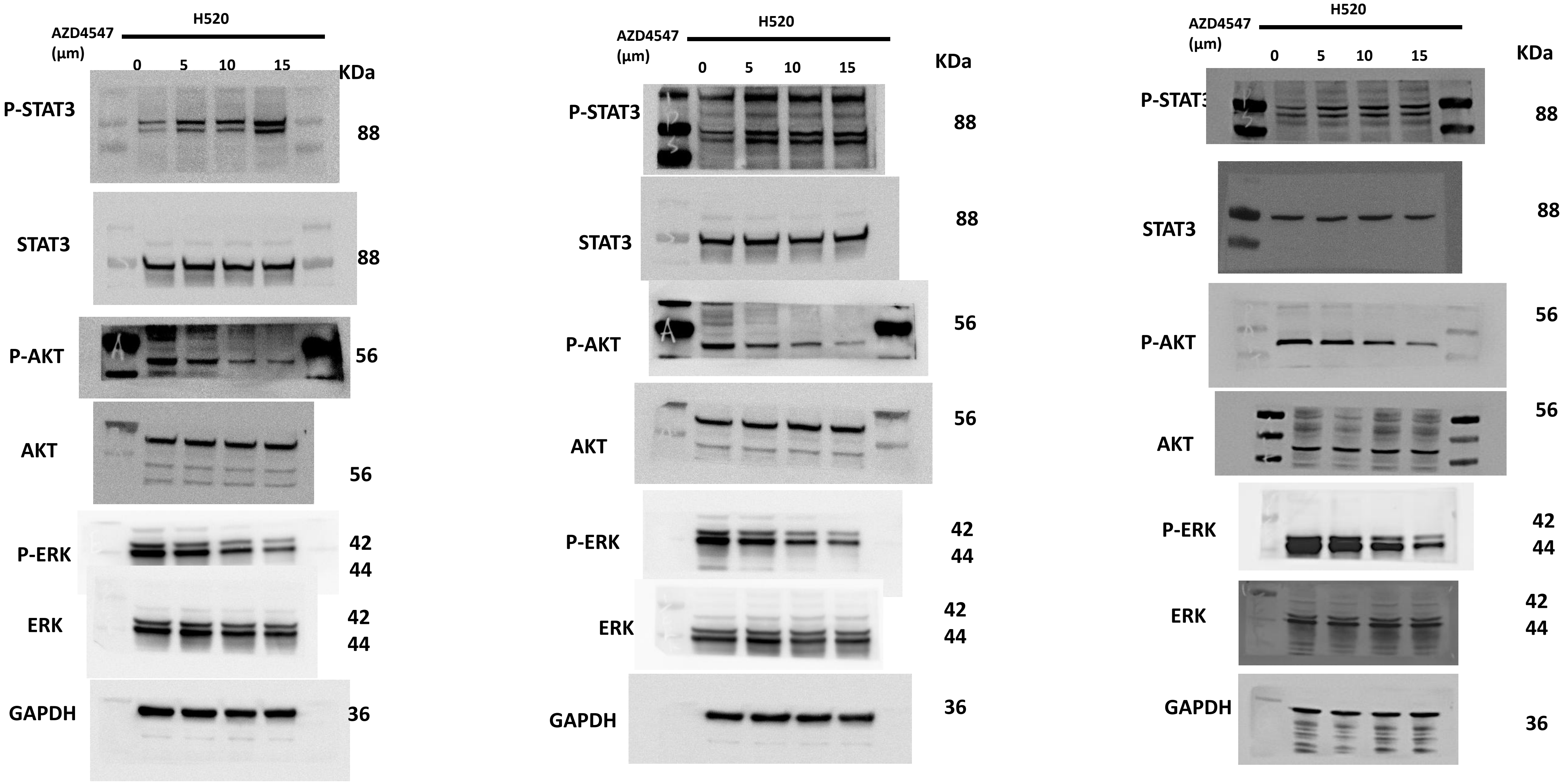

Uncropped gels for Westem Blots in Figure 1

F1-E(H1703)

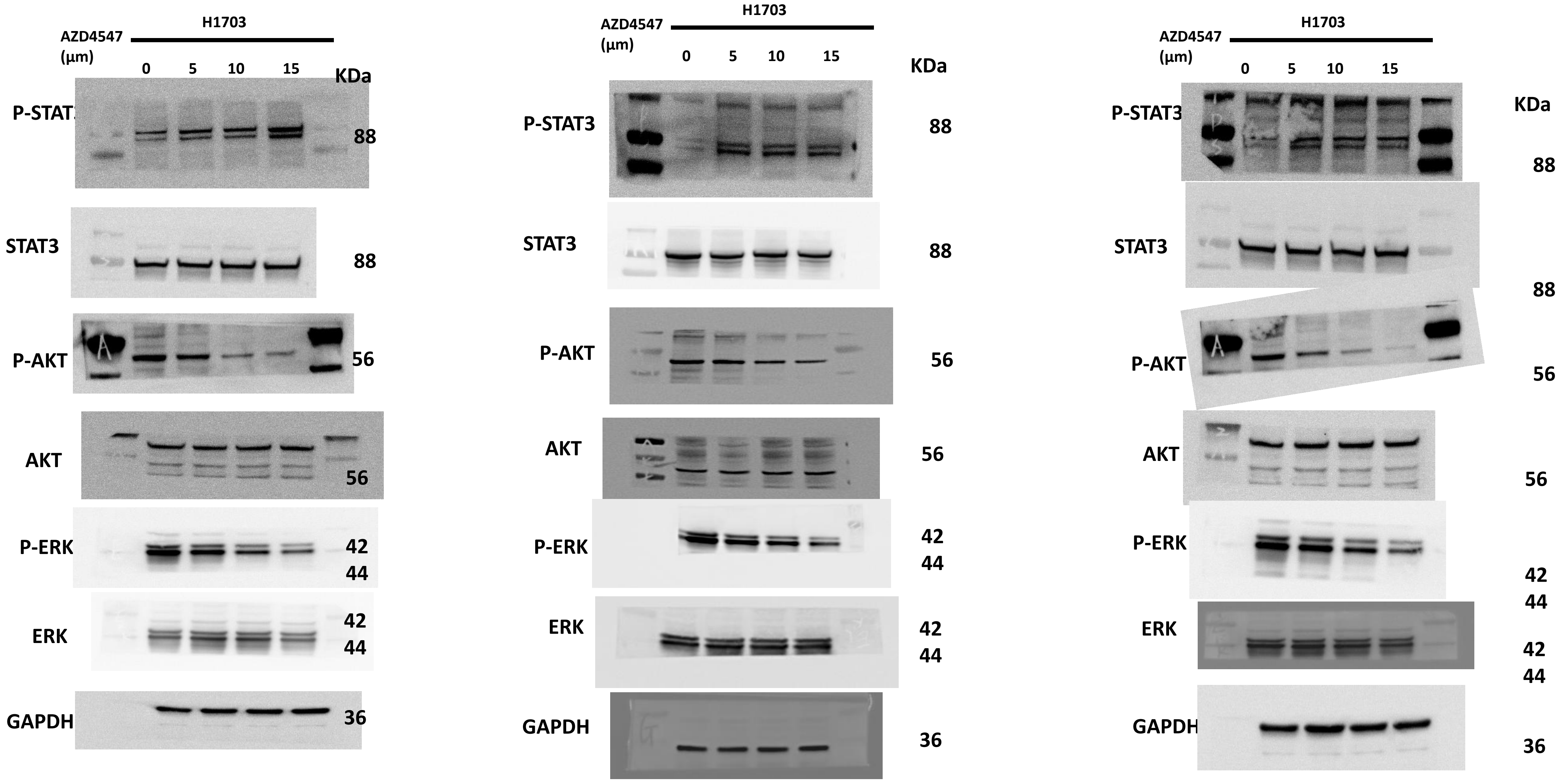

Uncropped gels for Westem Blots in Figure 1

F1-F

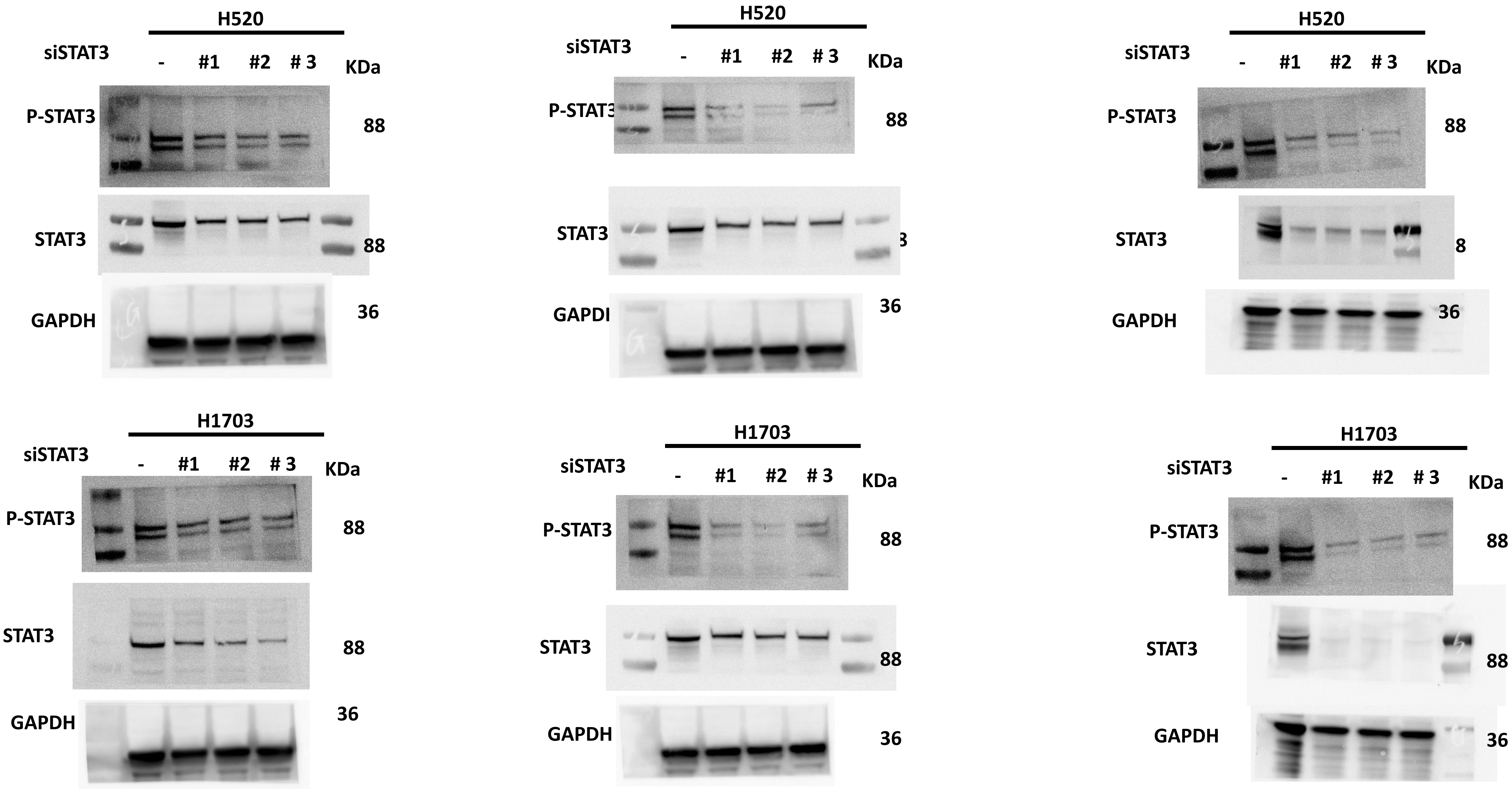

Uncropped gels for Westem Blots in Figure 1

F1-F

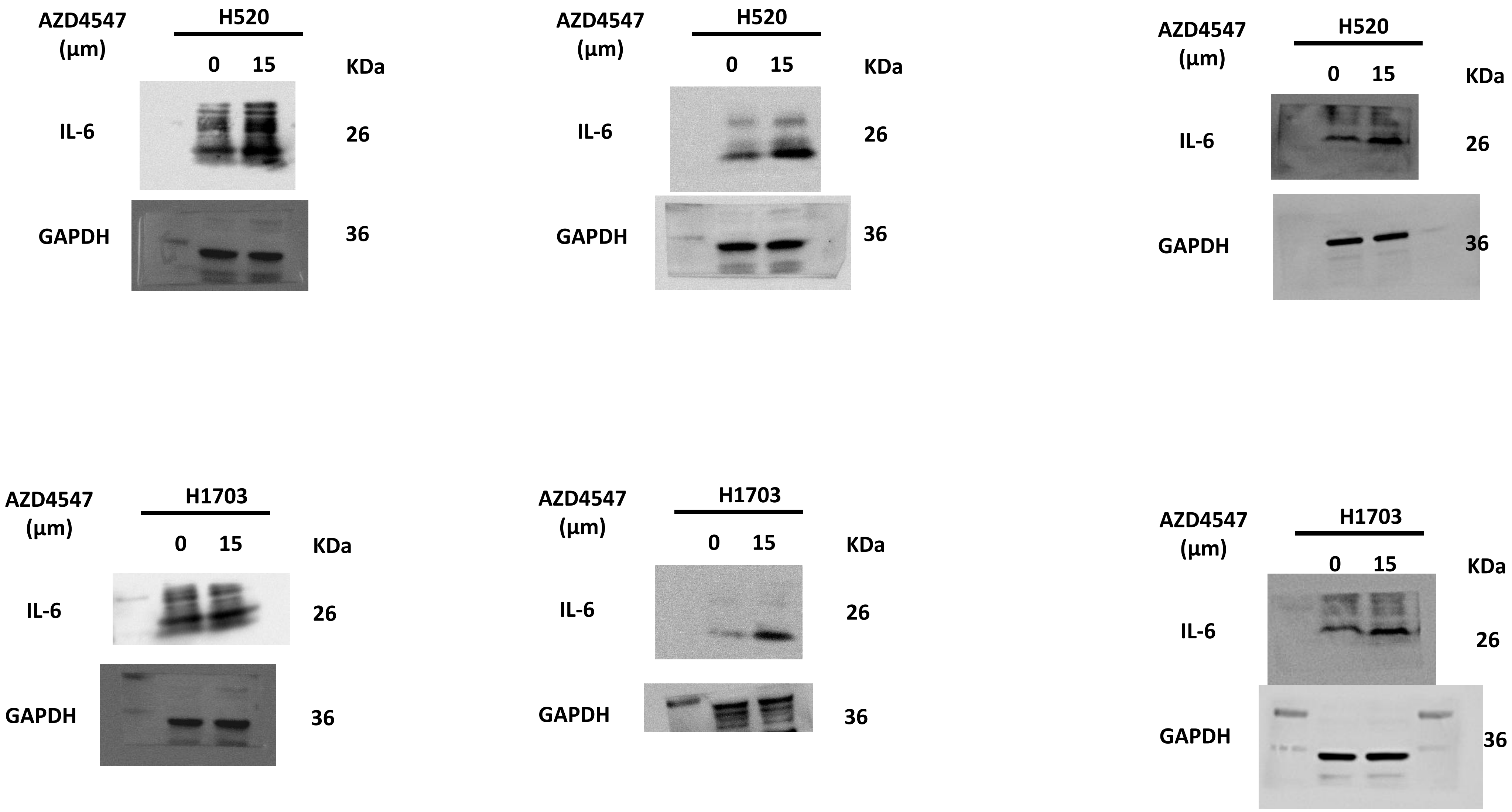

Uncropped gels for Westem Blots in Figure 1

F1-K

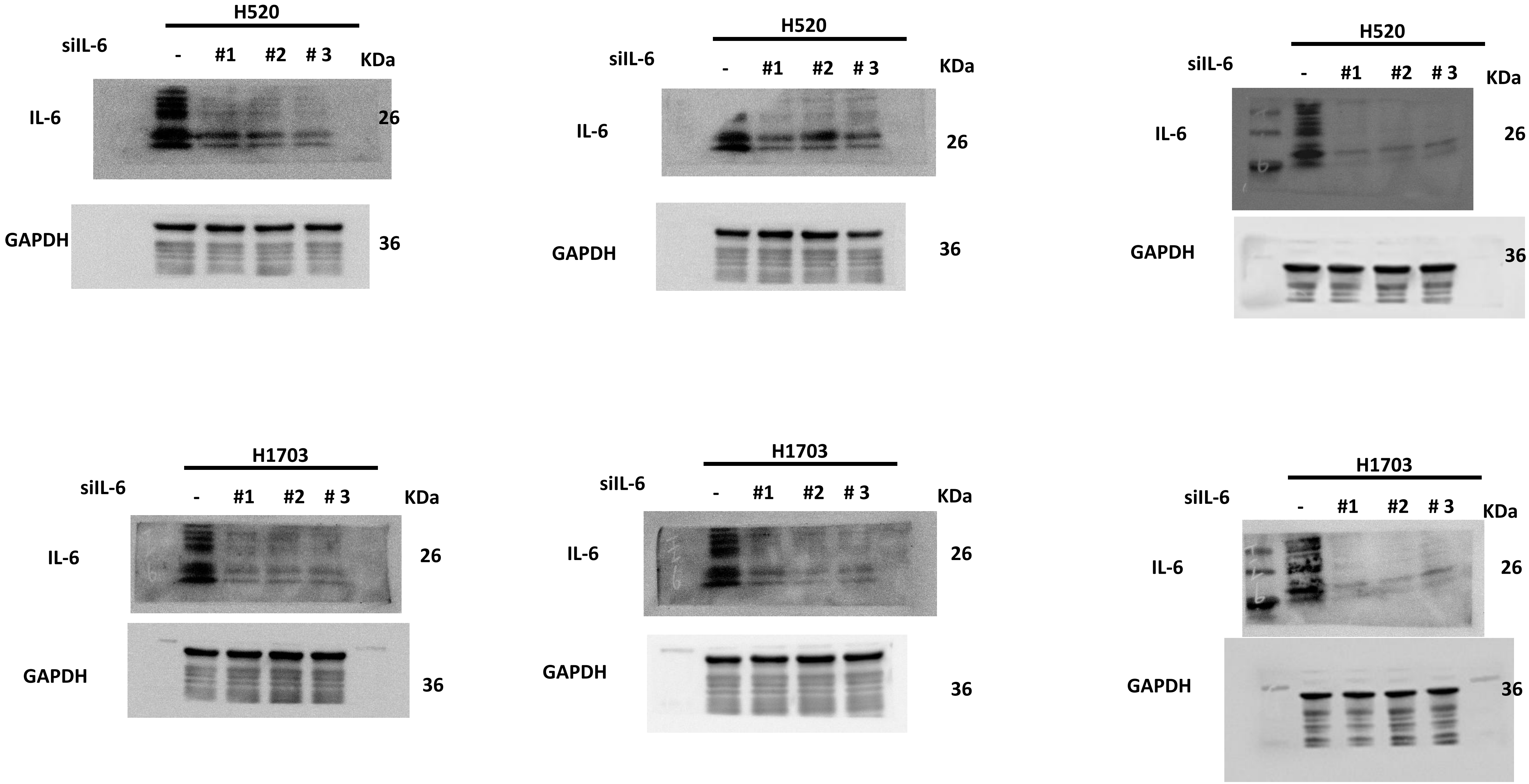

Uncropped gels for Westem Blots in Figure 1

F1-M

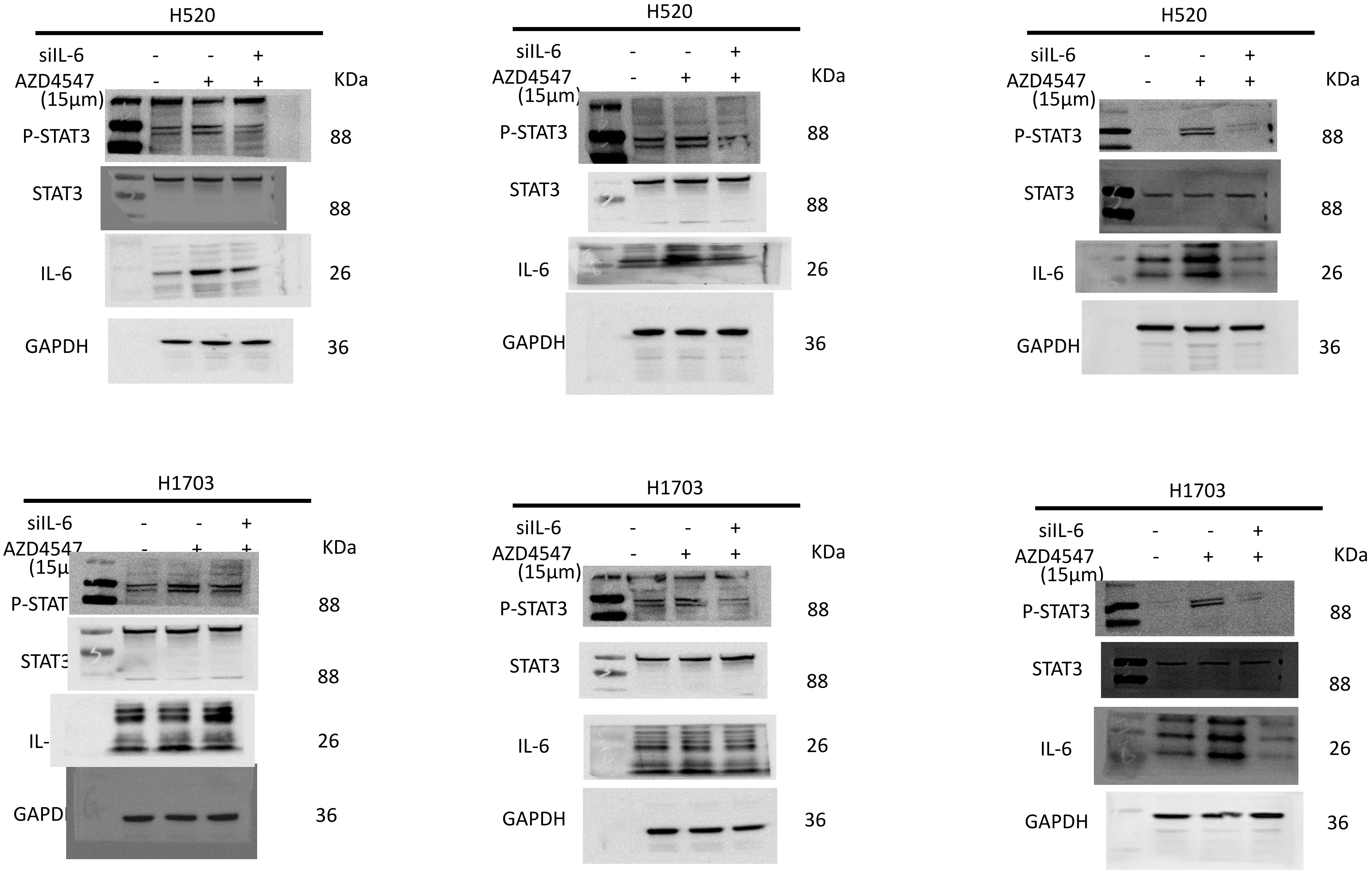

Uncropped gels for Westem Blots in Figure 2

F2-A

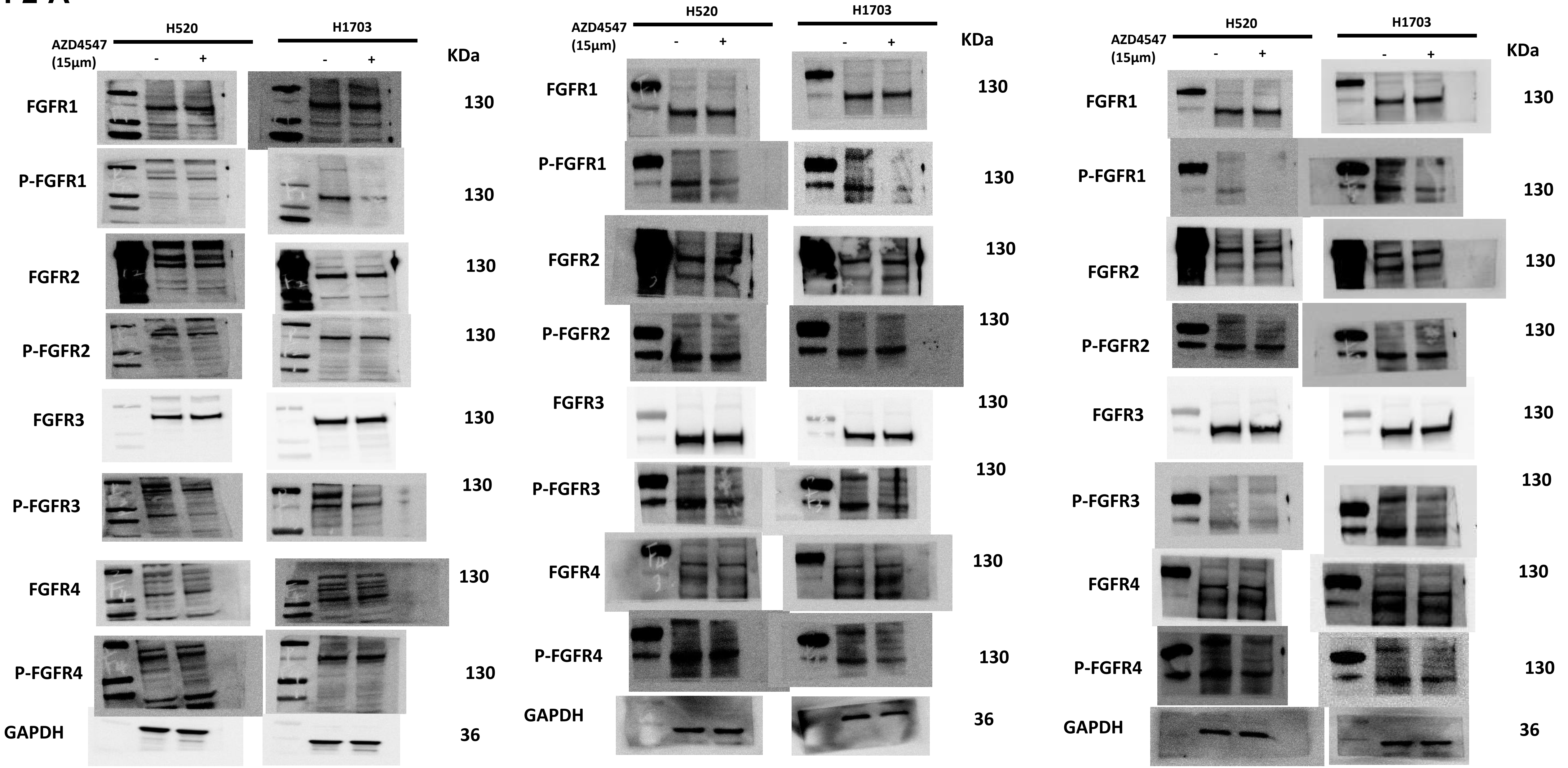

Uncropped gels for Westem Blots in Figure 2

F2-E

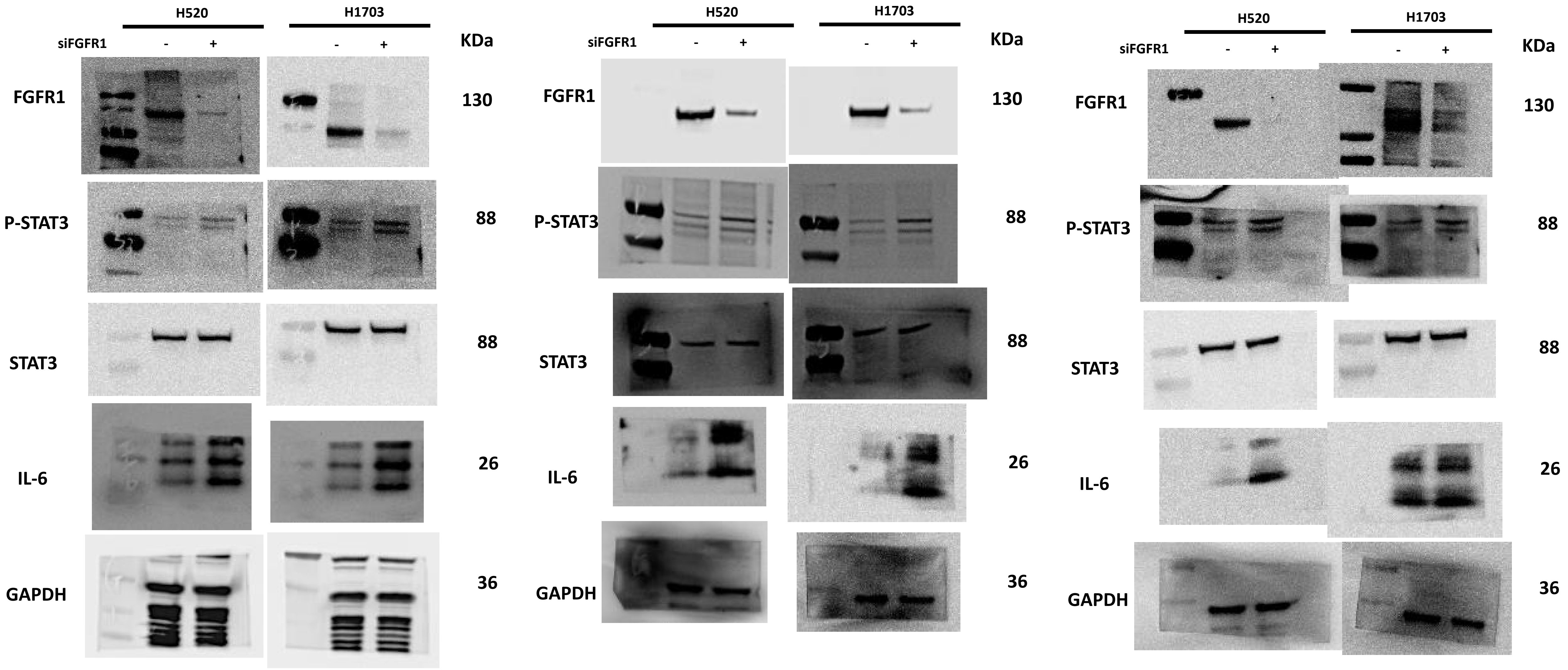

Uncropped gels for Westem Blots in Figure 2

F2-F

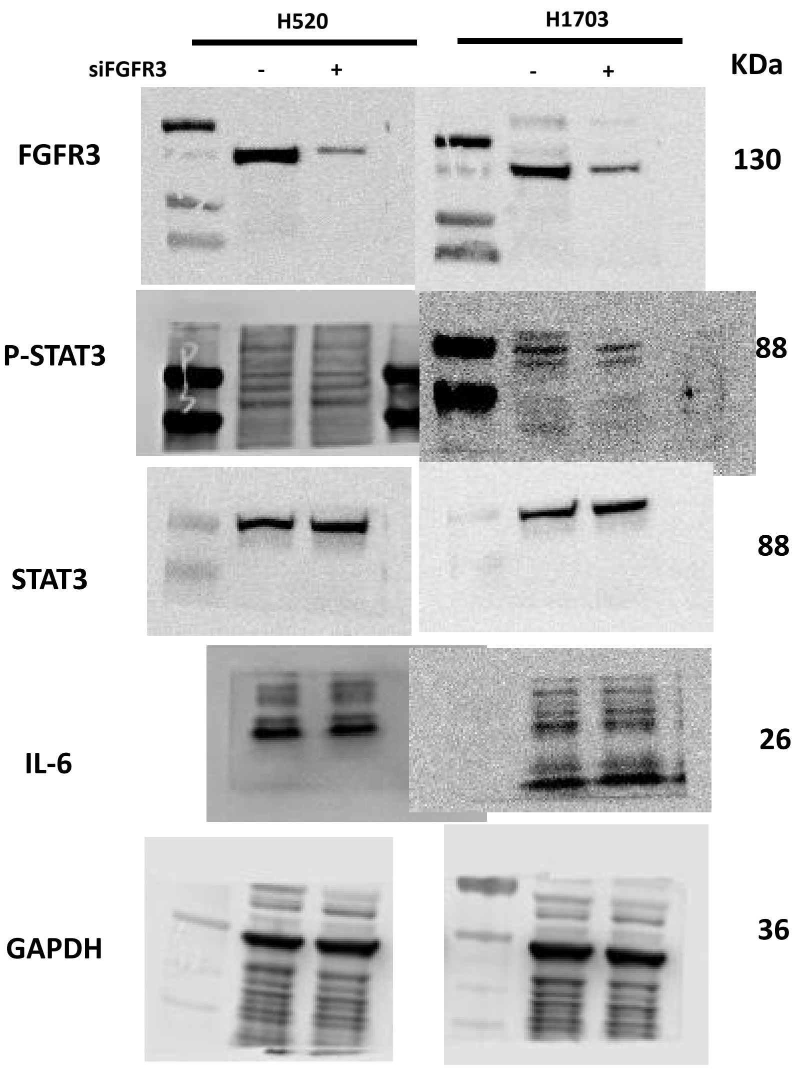

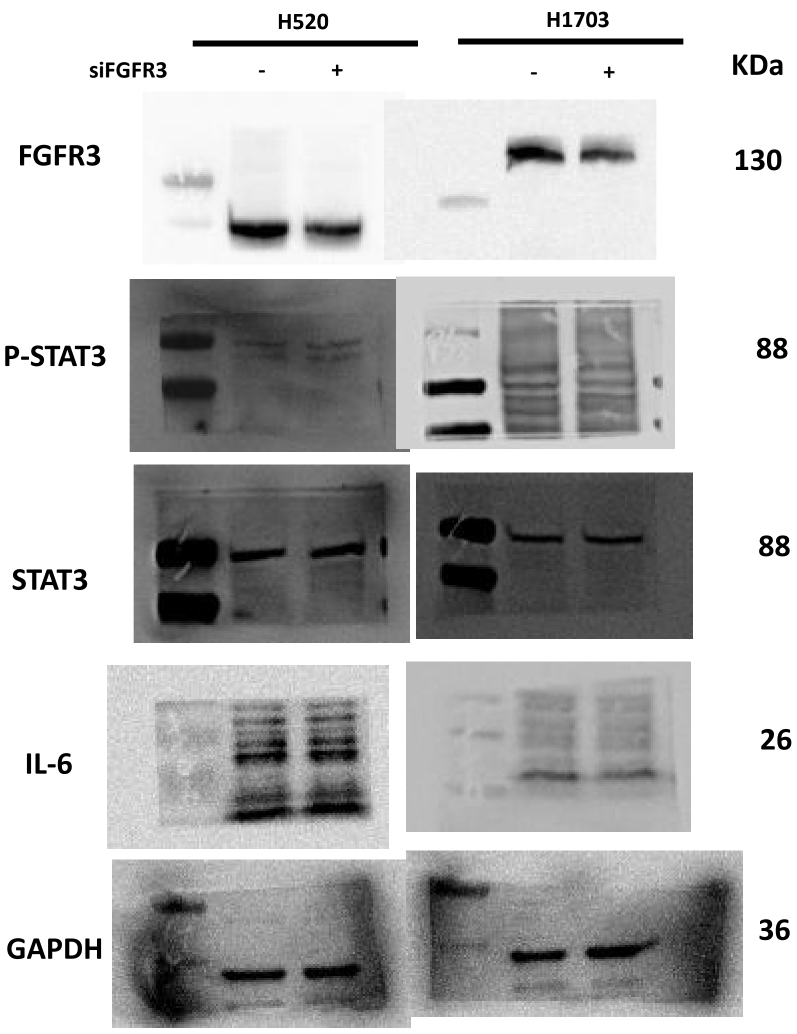

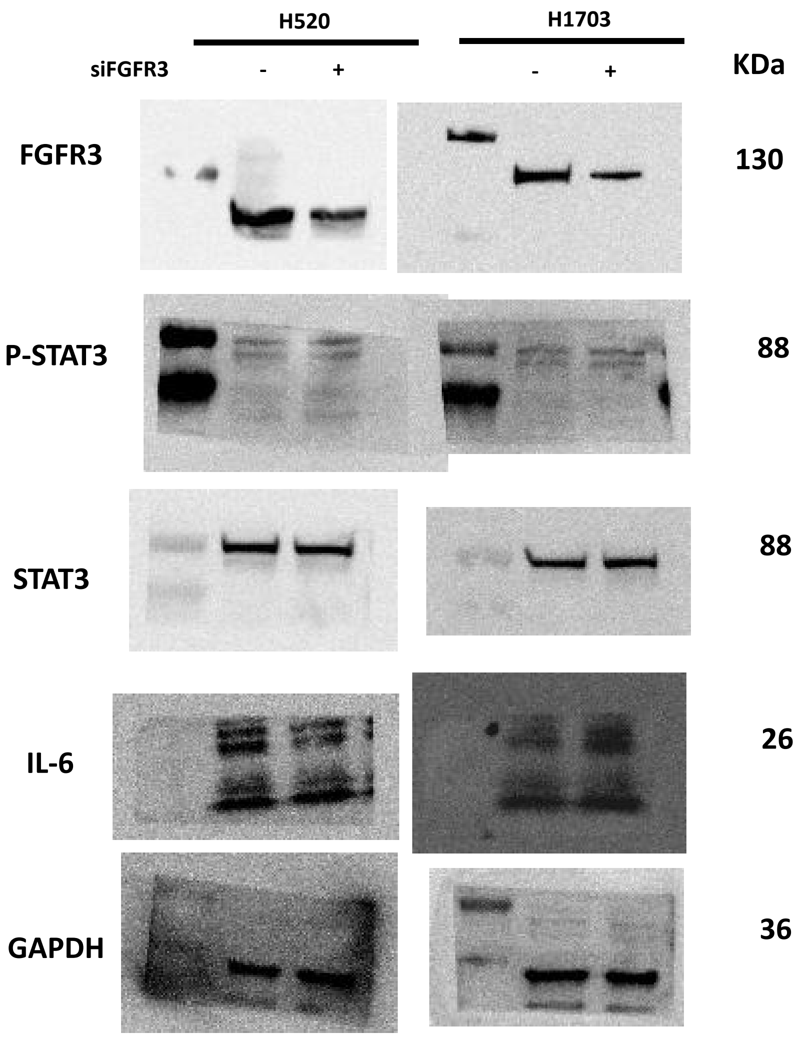

F3-G

Uncropped gels for Westem Blots in Figure 3

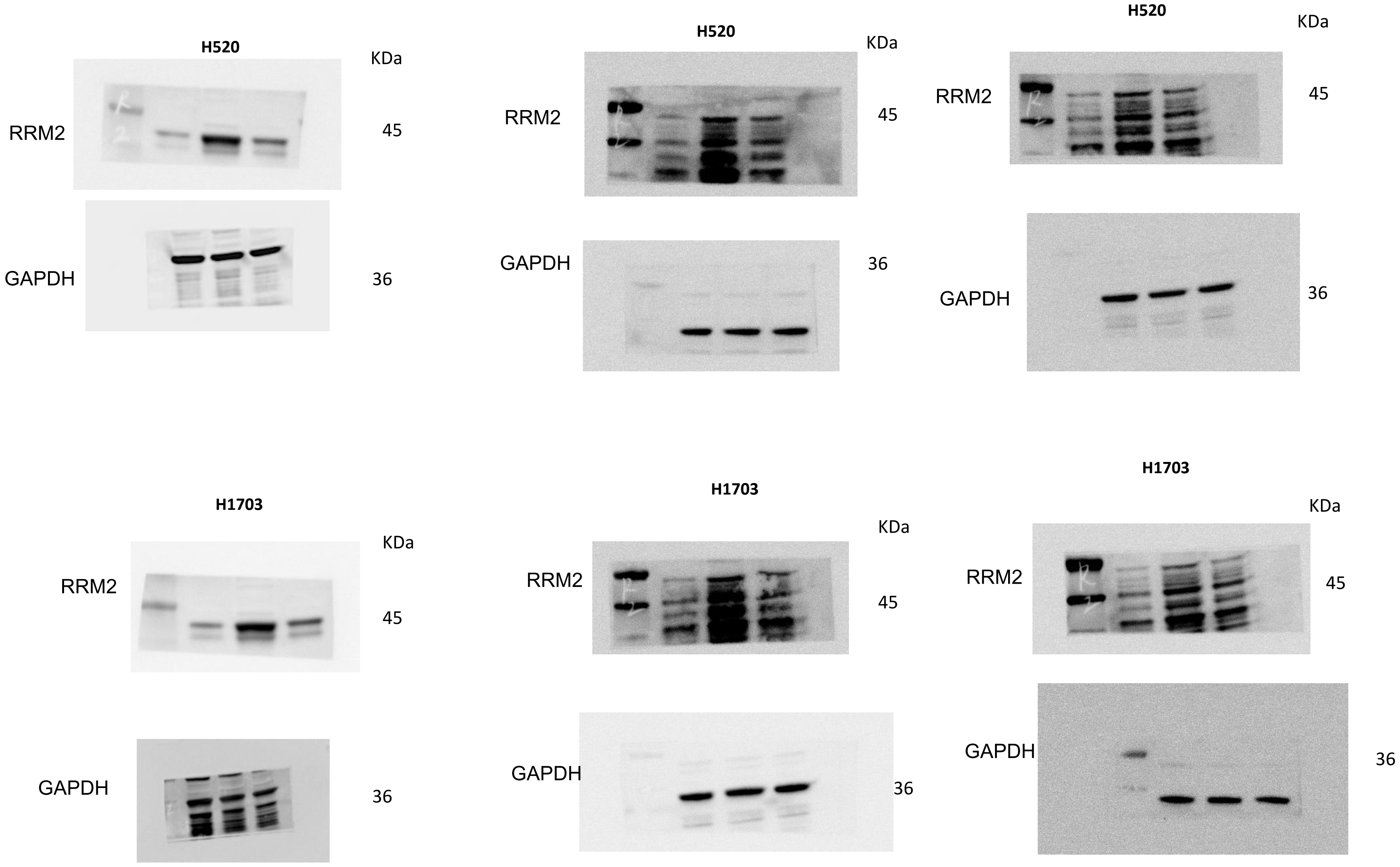

F3-K

Uncropped gels for Westem Blots in Figure 3

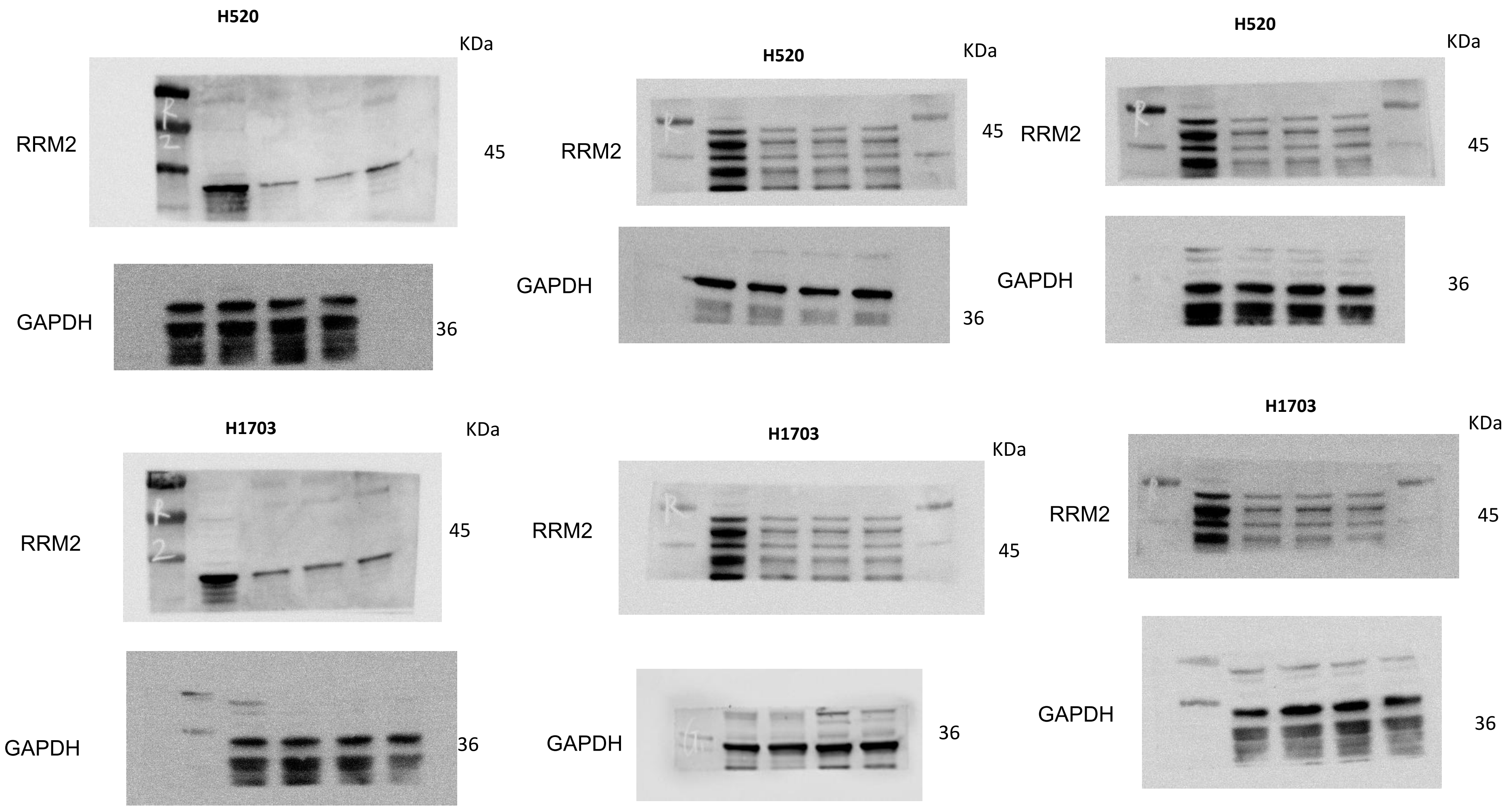

F3-M

Uncropped gels for Westem Blots in Figure 3

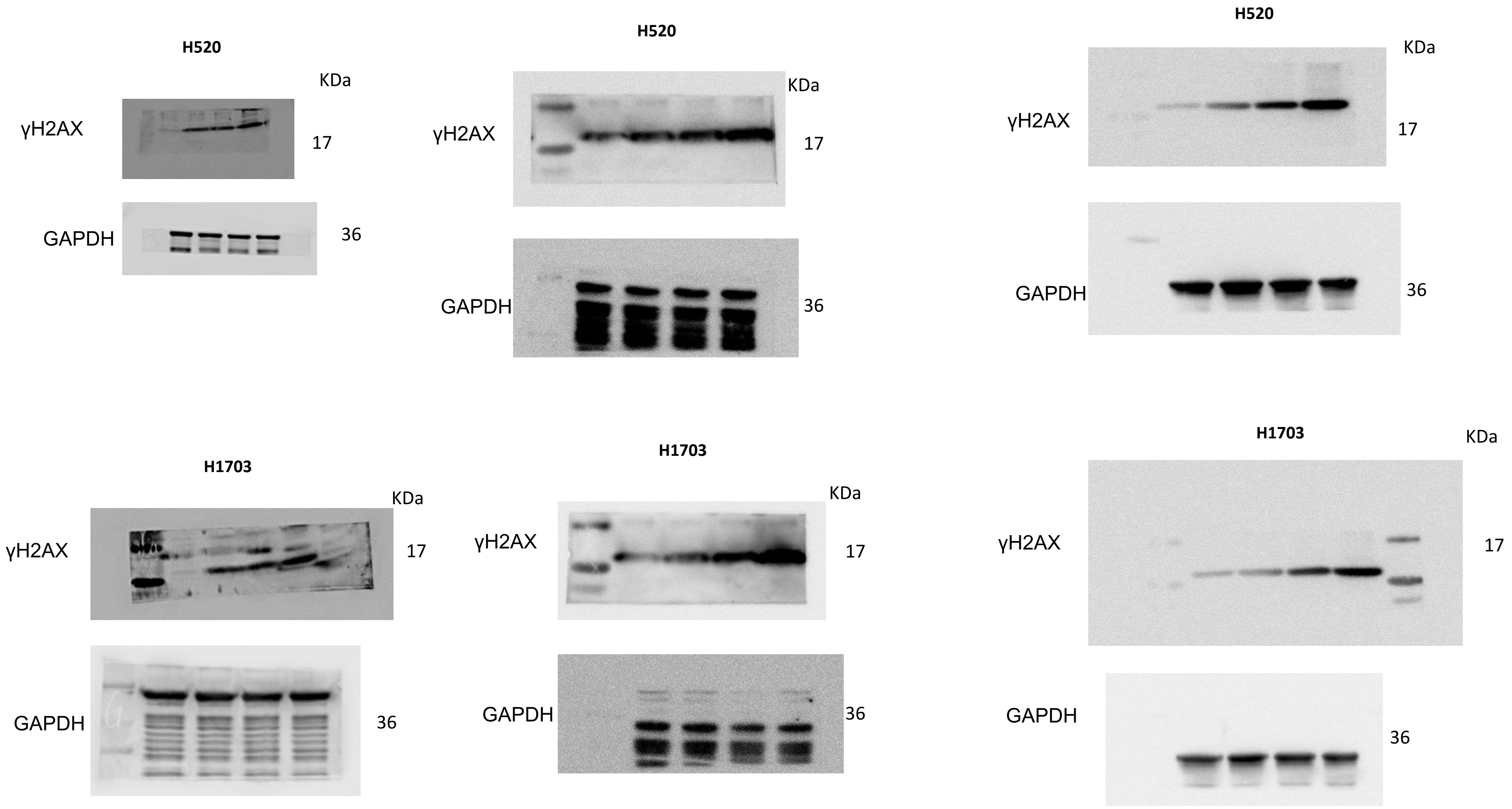

F4-F

Uncropped gels for Westem Blots in Figure 4

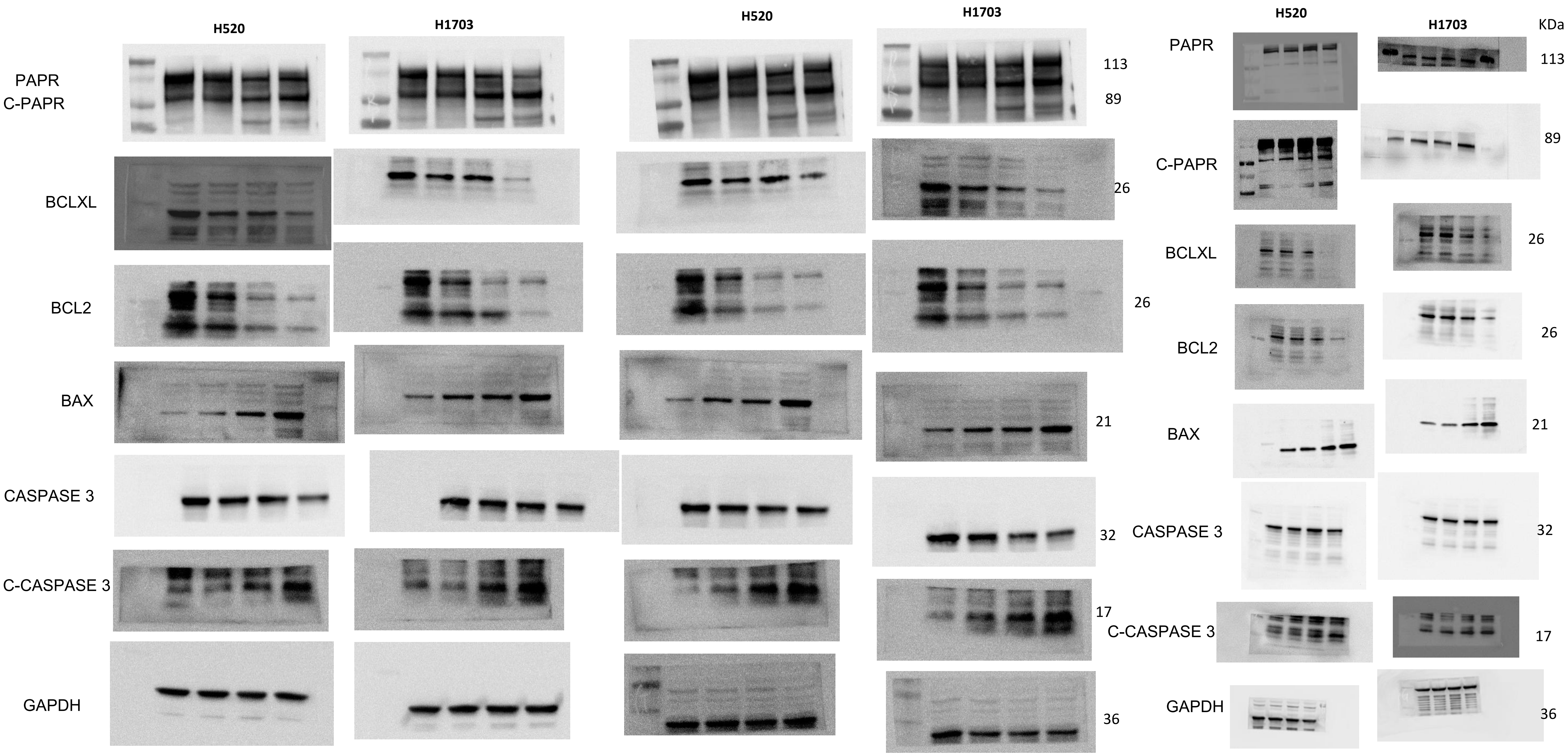

Uncropped gels for Westem Blots in Figure 5

F5-A

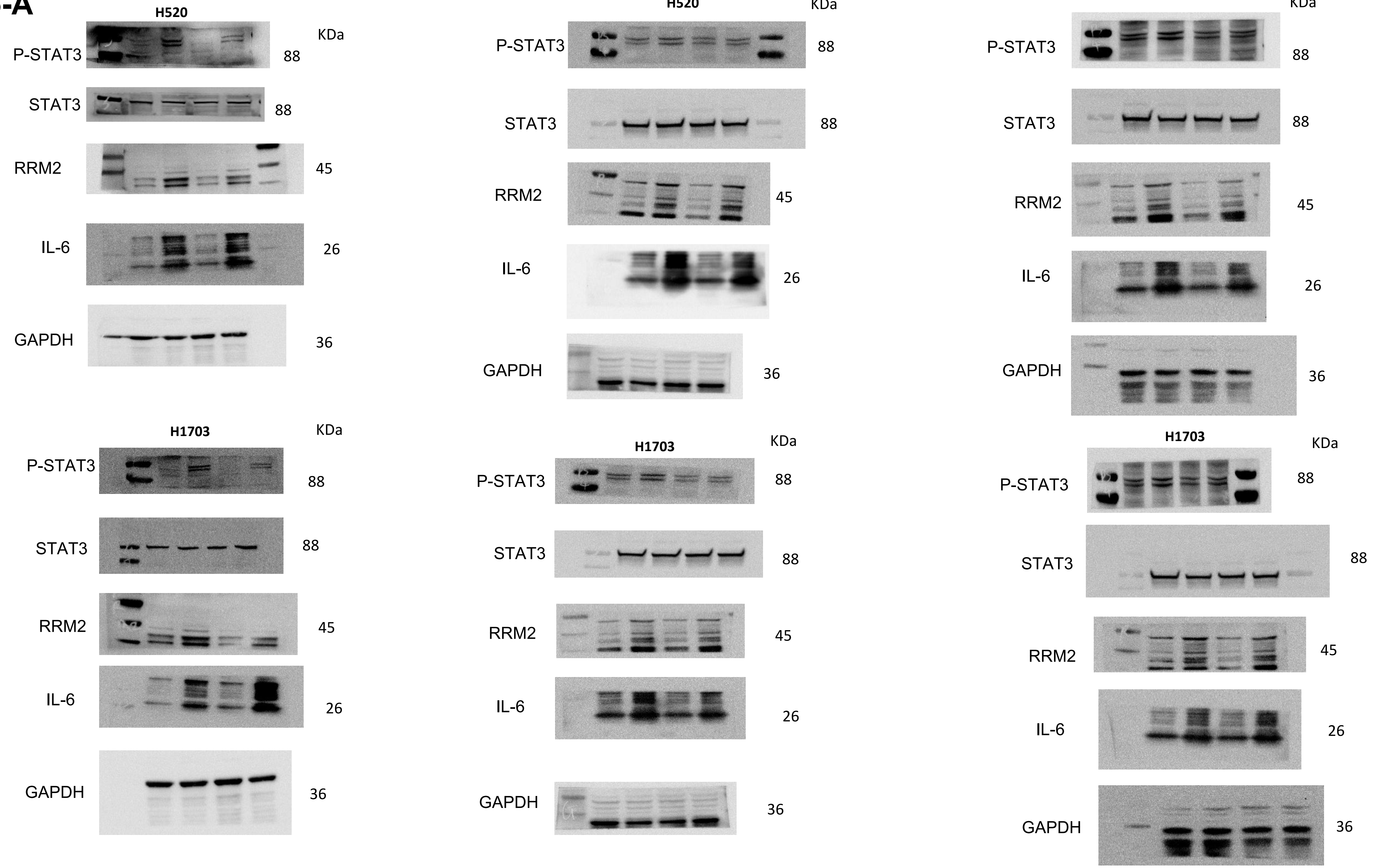

F5-B

Uncropped gels for Westem Blots in Figure 5

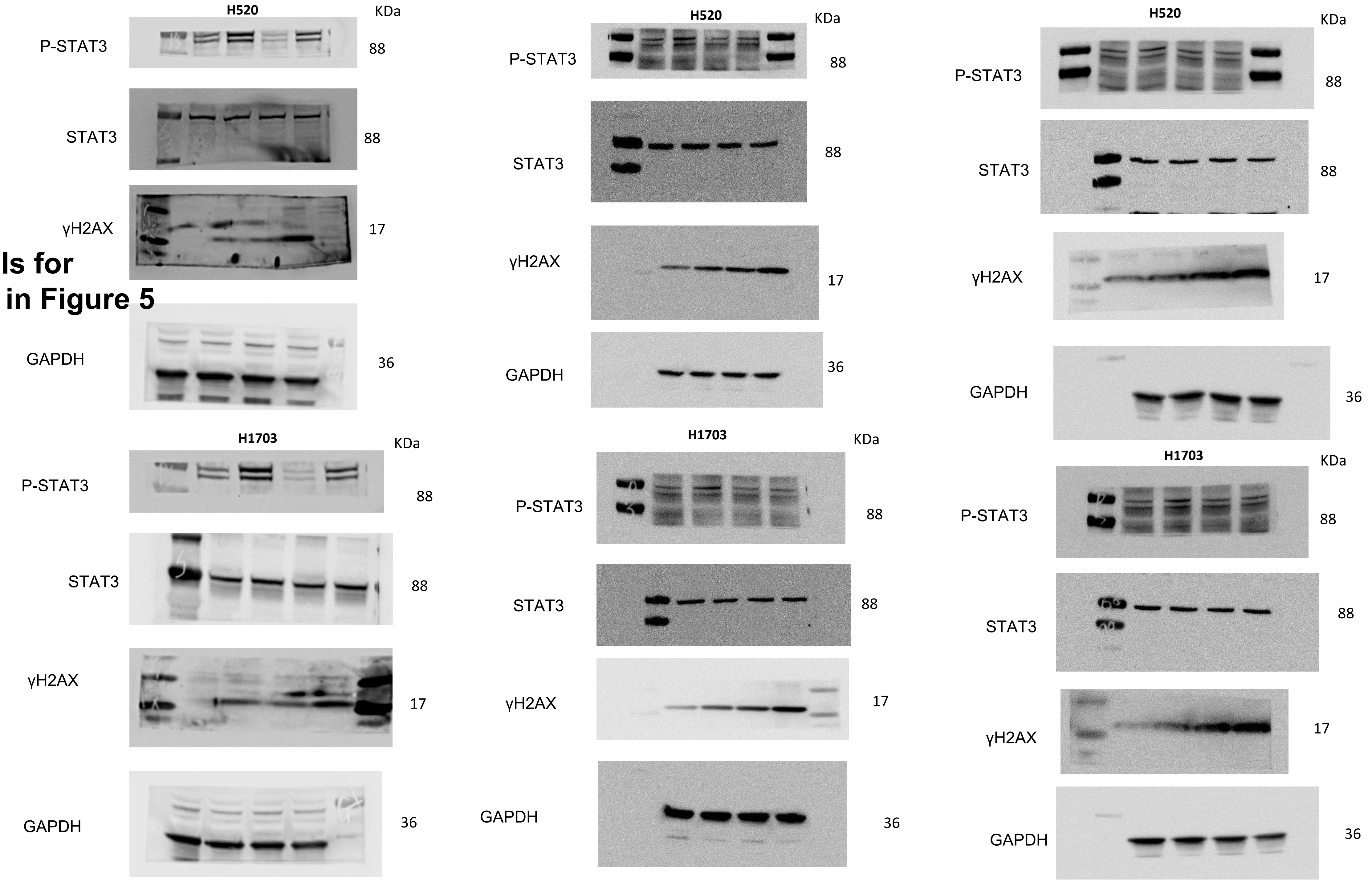

F5-D Uncropped gels for Westem Blots in Figure 5

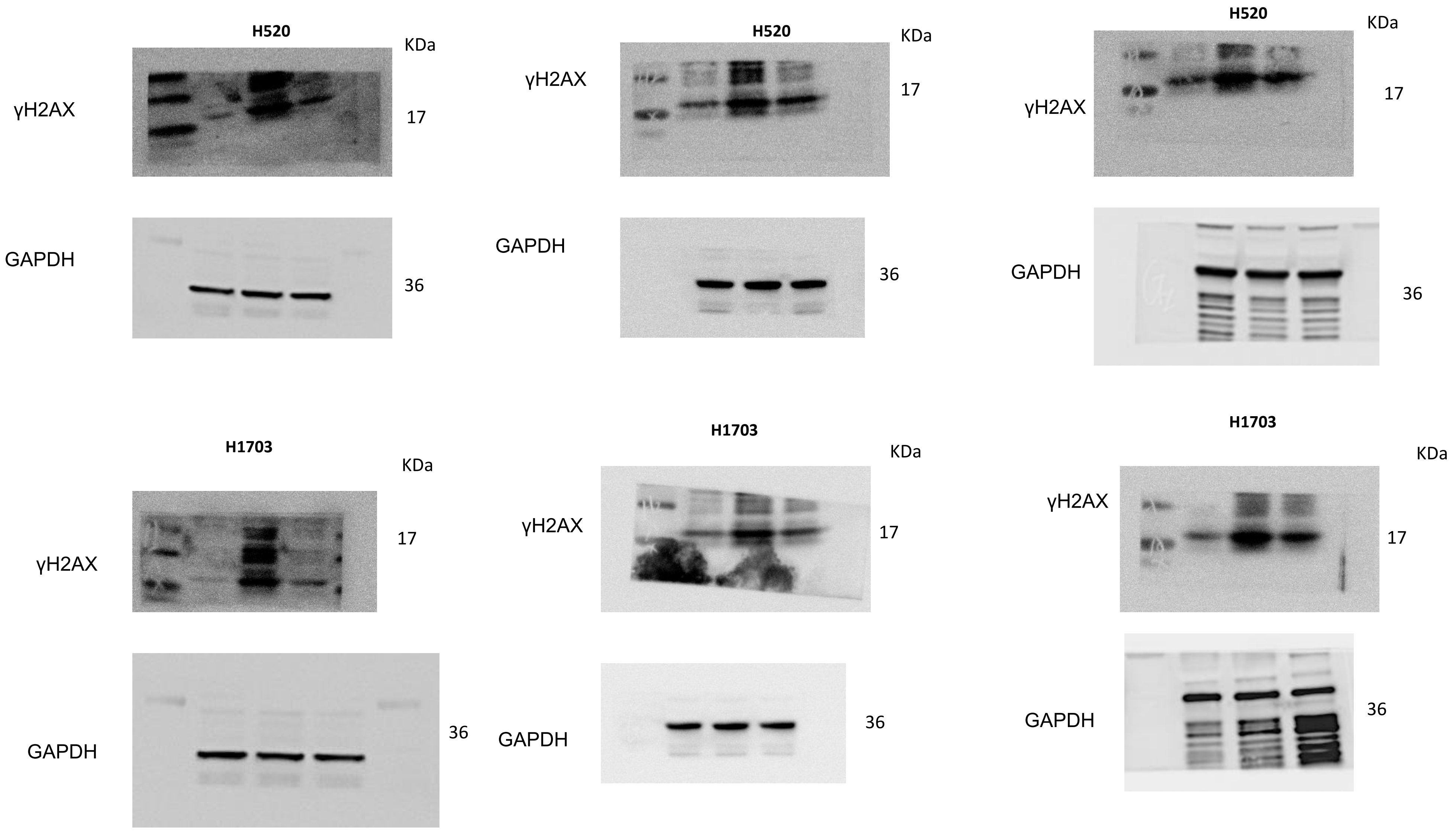

F5-E

Uncropped gels for Westem Blots in Figure 5

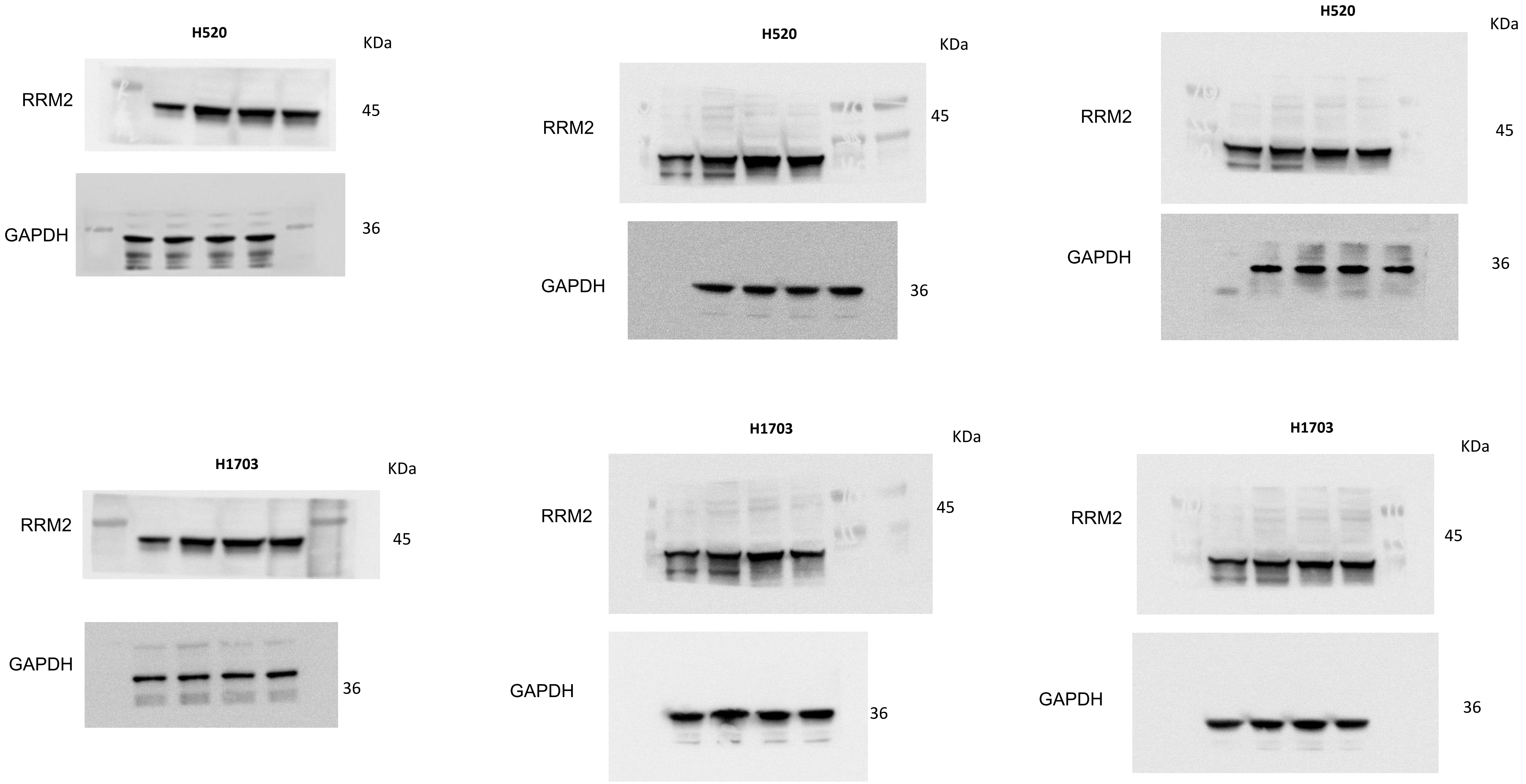

F5-H Uncropped gels for Westem Blots in Figure 5

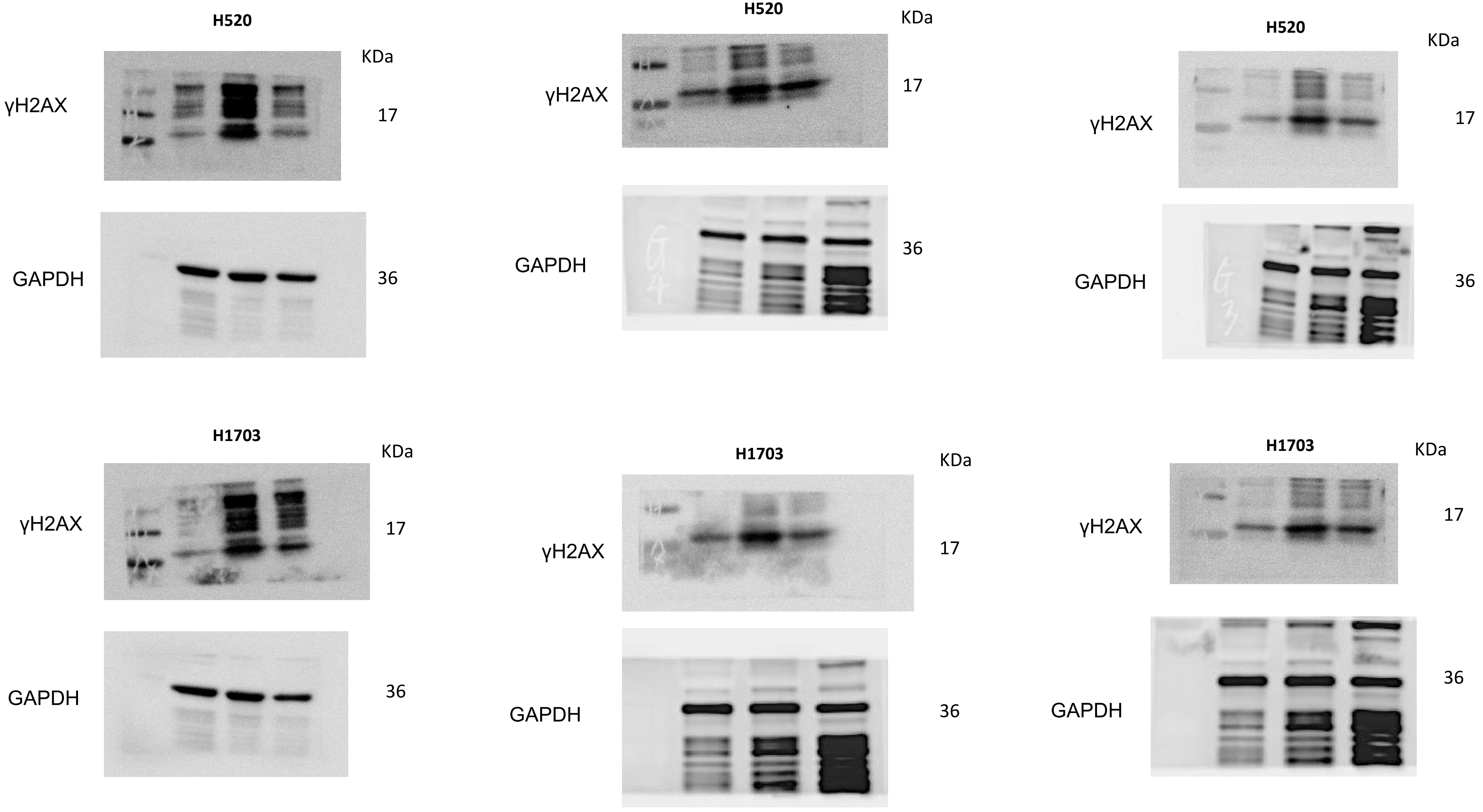

F7-E

Uncropped gels for Westem Blots in Figure 7

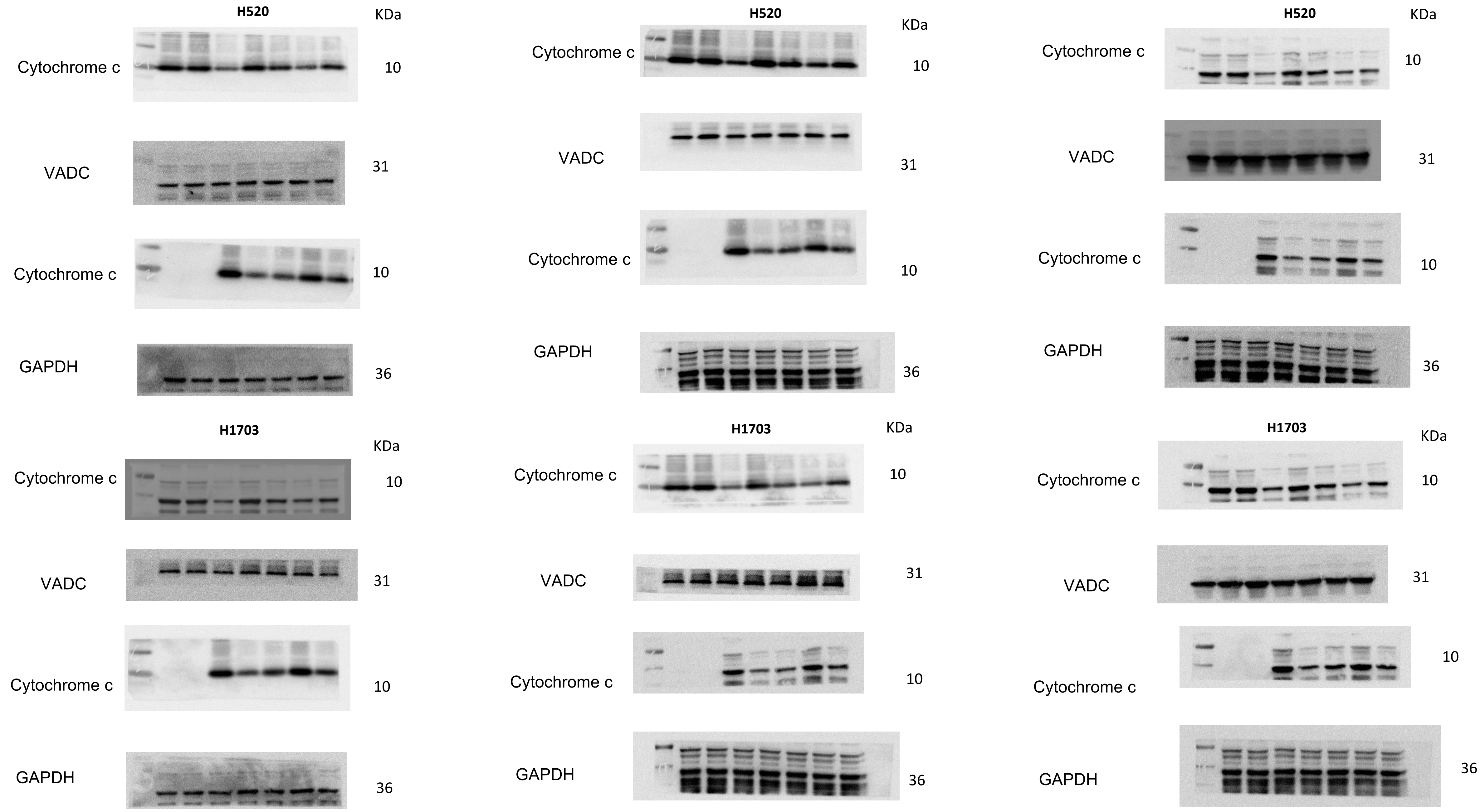

F1-B **Uncropped gels for Westem Blots in Figure 1**

	H520			H1703			BEAS-2B		
FGFR1	3.801	3.773	3.709	3.65	3.666	3.713	1	1	1
FGFR2	3.83	3.865	3.775	3.86	3.821	3.757	1	1	1
FGFR3	2.631	2.556	2.603	2.724	2.688	2.707	1	1	1
FGFR4	1.554	1.576	1.469	1.335	1.403	1.442	1	1	1

F1-E **Uncropped gels for Westem Blots in Figure 1**

H520

p-STAT3/STAT3				p-AKT/AKT				p-ERK/ERK			
1	2.492	2.624	5.42	1	0.853	0.422	0.203	1	0.806	0.653	0.341
1	2.531	2.535	5.844	1	0.725	0.326	0.165	1	0.845	0.632	0.404
1	2.424	2.562	7.553	1	0.821	0.409	0.234	1	0.829	0.529	0.323

H1703

p-STAT3/STAT3				p-AKT/AKT				p-ERK/ERK			
1	2.526	2.952	5.626	1	0.824	0.462	0.2452	1	0.806	0.521	0.251
1	2.626	2.782	5.245	1	0.811	0.466	0.265	1	0.813	0.551	0.352
1	2.2629	2.846	6.435	1	0.745	0.524	0.257	1	0.735	0.514	0.33

F1-F

Uncropped gels for Westem Blots in Figure 1

H520

STAT3			
-	#1	#2	#3
1	0.32	0.34	0.34
1	0.28	0.2	0.3
1	0.24	0.3	0.33

H1703

STAT3			
1	0.2	0.23	0.23
1	0.3	0.34	0.2
1	0.21	0.25	0.26

F1-J Uncropped gels for Westem Blots in Figure 1

H520		H1703	
IL-6		IL-6	
1	2.35	1	1.76
1	2.42	1	1.64
1	2.16	1	1.69

F1-K Uncropped gels for Westem Blots in Figure 1

H520				H1703			
IL-6				IL-6			
1	0.345	0.376	0.317	1	0.352	0.362	0.252
1	0.337	0.352	0.351	1	0.363	0.385	0.375
1	0.312	0.338	0.382	1	0.4	0.356	0.348

F1-M

Uncropped gels for Westem Blots in Figure 1

H520

pSTAT3/STAT3			IL-6		
1	3.42	0.86	1	3.25	1.41
1	4.26	1.23	1	2.24	0.98
1	5.34	1.32	1	3.13	1.21

H1703

pSTAT3/STAT3			IL-6		
1	2.97	1.42	1	2.43	1.21
1	3.63	1.27	1	2.36	1.05
1	3.15	1.295	1	1.89	0.87

F2-A

Uncropped gels for Westem Blots in Figure 2

H520

p-FGFR1/FGFR1		p-FGFR2/FGFR2		p-FGFR3/FGFR3		p-FGFR4/FGFR4	
1	0.51	1	1.14	1	0.65	1	1.12
1	0.45	1	1.01	1	0.57	1	1.14
1	0.5	1	0.86	1	0.69	1	1.21

H1703

p-FGFR1/FGFR1		p-FGFR2/FGFR2		p-FGFR3/FGFR3		p-FGFR4/FGFR4	
1	0.432	1	0.986	1	0.415	1	1.144
1	0.471	1	1.125	1	0.561	1	0.956
1	0.375	1	0.897	1	0.365	1	1.135

F2-E

Uncropped gels for Westem Blots in Figure 2

H520

FGFR1		pSTAT3/STAT3		IL-6	
**1**	**0.345**	**1**	**5.456**	**1**	**2.642**
**1**	**0.215**	**1**	**5.72**	**1**	**2.426**
**1**	**0.276**	**1**	**4.573**	**1**	**2.622**

H1703

FGFR1		pSTAT3/STAT3		IL-6	
1	0.231	1	6.42	1	2.153
1	0.272	1	6.252	1	2.626
1	0.221	1	5.732	1	2.362

Uncropped gels for Westem Blots in Figure 2

F2-F

H520

FGFR3		pSTAT3/STAT3		IL-6	
1	0.251	1	1.124	1	1.15
1	0.124	1	0.934	1	1.051
1	0.211	1	1.051	1	1.051

H1703

FGFR3		pSTAT3/STAT3		IL-6	
1	0.171	1	0.753	1	1.041
1	0.158	1	0.842	1	1.082
1	0.234	1	0.731	1	0.961

F3-G

Uncropped gels for Westem Blots in Figure 3

H520			H1703		
RRM2			RRM2		
1	4.72	1.36	1	5.31	1.78
1	3.46	1.54	1	5.15	1.89
1	4.34	1.58	1	4.65	2.01

F3-K

Uncropped gels for Westem Blots in Figure 3

H520				H1703			
RRM2				RRM2			
1	0.4	0.46	0.51	1	0.32	0.35	0.35
1	0.45	0.52	0.415	1	0.43	0.31	0.36
1	0.45	0.43	0.44	1	0.47	0.37	0.38

F3-M

Uncropped gels for Westem Blots in Figure 3

H520				H1703			
γ-H2AX				γ-H2AX			
1	1.42	1.85	2.15	1	1.306	1.53	2.82
1	1.46	1.65	2.35	1	1.426	1.73	2.62
1	1.51	1.75	2.62	1	1.46	1.73	2.46

F4-F **H520** **Uncropped gels for Westem Blots in Figure 4**

cleaved PAPR				BCLXL				BCL2				BAX				Cleaved caspase			
1	1.23	2.54	3.34	1	0.84	0.45	0.23	1	0.56	0.5	0.32	1	1.5	2.3	4.35	1	1.12	1.23	2.41
1	1.14	2	3.56	1	0.7	0.42	0.26	1	0.58	0.47	0.25	1	1.42	2.24	4.98	1	1.09	1.34	2.53
1	1.25	2.15	3.39	1	0.77	0.52	0.26	1	0.57	0.43	0.4	1	1.45	2.11	4.31	1	1.05	1.31	2.65

H1703

cleaved PAPR				BCLXL				BCL2				BAX				Cleaved caspase			
1	1.27	2.54	4.63	1	0.74	0.56	0.21	1	0.64	0.67	0.35	1	2.4	2.34	4.73	1	1.64	1.36	2.26
1	1.28	2	4.56	1	0.72	0.63	0.2	1	0.58	0.65	0.38	1	2.5	2.51	4.08	1	1.25	1.73	2.46
1	1.21	2.15	3.98	1	0.75	0.58	0.24	1	0.63	0.58	0.43	1	2.35	2.16	4.71	1	1.16	1.77	2.75

F5-A

H520

Uncropped gels for Westem Blots in Figure 5

pSTAT3/STAT3				RRM2				IL-6			
1	3.32	0.32	1.22	1	4.12	0.98	1.64	1	5.31	1.24	6.23
1	3.12	0.56	1.06	1	3.42	1.39	1.52	1	5.31	1.42	6.24
1	3	0.59	1.39	1	3.52	0.82	1.74	1	4.51	1.15	5.82

H1703

pSTAT3/STAT3				RRM2				IL-6			
1	3.45	0.42	1.35	1	4.27	0.86	1.41	1	5.43	1.21	6.45
1	3.26	0.68	1.19	1	3.58	0.73	1.47	1	5.88	1.13	7.28
1	3.17	0.72	1.53	1	3.69	0.94	1.27	1	4.66	1.04	7.45

F5-B

Uncropped gels for Westem Blots in Figure 5

H520				H1703			
γ-H2AX				γ-H2AX			
1	1.4	1.64	2.31	1	1.51	1.52	2.14
1	1.45	1.68	2.35	1	1.47	1.39	2.34
1	1.47	1.47	2.23	1	1.37	1.55	2.38

F5-D

Uncropped gels for Westem Blots in Figure 5

H520			H1703		
γ-H2AX			γ-H2AX		
1	3.51	1.53	1	5.32	1.23
1	3.25	1.35	1	6.31	1.65
1	3.76	1.51	1	3.45	1.53

F5-G

Uncropped gels for Westem Blots in Figure 5

H520			H1703		
γ-H2AX			γ-H2AX		
1	5.72	1.64	1	5.16	2.73
1	4.24	1.52	1	6.26	2.62
1	5.53	1.77	1	5.73	2.57

F7-E

Uncropped gels for Westem Blots in Figure 7

H520							H1703						
Cytochrome							Cytochrome						
1	1	0.23	0.81	0.62	0.24	0.57	1	1.21	0.43	1.21	0.73	0.35	0.63
1	1.1	0.25	0.76	0.56	0.32	0.54	1	1.15	0.36	0.86	0.72	0.36	0.56
1	1.15	0.2	0.8	0.57	0.35	0.45	1	1.13	0.38	0.92	0.79	0.38	0.58
